# Unfolded Protein Response and Scaffold Independent Pheromone MAP Kinase Signaling Control *Verticillium dahliae* Growth, Development, and Plant Pathogenesis

**DOI:** 10.3390/jof7040305

**Published:** 2021-04-15

**Authors:** Jessica Starke, Rebekka Harting, Isabel Maurus, Miriam Leonard, Rica Bremenkamp, Kai Heimel, James W. Kronstad, Gerhard H. Braus

**Affiliations:** 1 Department of Molecular Microbiology and Genetics, Institute of Microbiology and Genetics and Göttingen Center for Molecular Biosciences (GZMB), University of Göttingen, 37077 Göttingen, Germany; jstarke@gwdg.de (J.S.); rhartin@gwdg.de (R.H.); isabel.maurus@uni-goettingen.de (I.M.); mleonar@gwdg.de (M.L.); rica.bremenkamp@uni-goettingen.de (R.B.); kheimel@gwdg.de (K.H.); 2 Michael Smith Laboratories, Department of Microbiology and Immunology, University of British Columbia, Vancouver, BC V6T 1Z4, Canada; kronstad@msl.ubc.ca

**Keywords:** *Verticillium dahliae*, plant pathogen, unfolded protein response (UPR), oleate ∆12-fatty acid desaturase, mitogen-activated protein kinase (MAPK) signaling, MAPK scaffold, dual-specificity MAPK phosphatase

## Abstract

Differentiation, growth, and virulence of the vascular plant pathogen *Verticillium dahliae* depend on a network of interconnected cellular signaling cascades. The transcription factor Hac1 of the endoplasmic reticulum-associated unfolded protein response (UPR) is required for initial root colonization, fungal growth, and vascular propagation by conidiation. Hac1 is essential for the formation of microsclerotia as long-time survival resting structures in the field. Single endoplasmic reticulum-associated enzymes for linoleic acid production as precursors for oxylipin signal molecules support fungal growth but not pathogenicity. Microsclerotia development, growth, and virulence further require the pheromone response mitogen-activated protein kinase (MAPK) pathway, but without the Ham5 scaffold function. The MAPK phosphatase Rok1 limits resting structure development of *V.* *dahliae*, but promotes growth, conidiation, and virulence. The interplay between UPR and MAPK signaling cascades includes several potential targets for fungal growth control for supporting disease management of the vascular pathogen *V.* *dahliae*.

## 1. Introduction

*Verticillium dahliae* is a soil-borne asexual ascomycete causing vascular wilt disease in a broad range of plants including high value crops [1,2]. Unfavorable conditions induce the formation of highly resistant, black melanized microsclerotia as characteristic dormant structures which persist in the soil and can overwinter for at least 14 years [1,3,4]. The fungus germinates upon recognition of an appropriate host and enters the plant preferably via natural root wounds, root tips, or lateral root hairs [5,6,7,8]. Hyphae grow from cortical cells towards the central cylinder and some of them successfully reach the xylem. In the vascular system, asexual spores spread via the transpiration stream [9]. Colonization of tissues neighboring the xylem correlates with induction of disease symptoms [10]. Limited nutrient availability in the dying host or in plant debris induces the formation of microsclerotia for persistence [10]. During the infection cycle, signals from the host environment induce differentiated fungal development and activate specific responses to enable colonization and suppression of the plant immune system. For the developmental response in fungi to external and internal signals, perception through receptors and signal transduction by different highly controlled signaling cascades is required. In consequence, these pathways lead to adaptations in transcription, translation, post-translational histone modifications, and protein stability [11].

The unfolded protein response (UPR) pathway is a control mechanism, which responds to internal stimuli by monitoring protein folding and secretion capacity in the endoplasmic reticulum (ER) lumen. It mediates expression of genes involved in ER stress relief [12,13,14,15]. Un- or misfolded proteins in the ER lumen are perceived either by direct interaction with transmembrane receptors such as yeast Ire1 or by interaction of these proteins with the heat shock protein Bip1/Kar2, which dissociates from Ire1 [16,17,18]. The cytoplasmic endoribonuclease domain of Ire1 is responsible for unconventional splicing of the mRNA encoding the basic leucine zipper (bZIP) transcription factor Hac1 [19,20]. Splicing of the uninduced *HAC1* (*HAC1^u^*) mRNA results in the induced *HAC1* (*HAC1^i^*) mRNA variant. The *HAC1^i^* mRNA is translated into the Hac1 protein, which regulates UPR target genes encoding, for example, regulators for adaptation of ER size, chaperones, glycosylation enzymes, and proteins required for vesicle transport or lipid biosynthesis [14,21,22,23,24,25,26].

The role of the UPR transcription factor Hac1 homologs and orthologues varies within fungal species pathogenic towards human or plants [27]. The opportunistic human pathogenic yeast *Candida glabrata* regulates ER stress responses independently of Hac1 via an Ire1-dependent decay of multiple ER-associated mRNAs [15,28,29]. Hemibiotrophic plant ascomycetes, such as the rice blast fungus *Magnaporthe oryzae*, require Hac1 and the UPR for conidia formation as well as for penetration and growth of invasive hyphae during plant infection [30]. In the Brassicaceae pathogen *Alternaria brassicicola*, the UPR is required for resistance against plant antimicrobial compounds and virulence, but is not involved in plant penetration and initial colonization [31,32]. The UPR pathway regulates virulence-specific genes in the dimorphic basidiomycete corn smut fungus *Ustilago maydis* [33,34,35].

Activation of the UPR results in adaptation of the ER size, which correlates with the increase of ER volume and biogenesis of membranes [36,37]. In yeast, genes involved in lipid metabolism are upregulated in response to an active UPR [25]. In mycelia of several fungal species, the polyunsaturated fatty acid linoleic acid (18:2Δ9,12) is an important membrane component and the major precursor of fungal oxygenated polyunsaturated fatty acids (oxylipins) [38,39,40,41,42]. Linoleic acid is synthesized by oleate Δ12-fatty acid desaturases localized to ER membranes, such as OdeA that acts on oleic acid (18:1Δ9) by introduction of a second double bond into the carbon chain at position 12 from the carboxy-terminus [43]. Oxylipin precursor synthesis via OdeA is required for asexual and sexual development as well as for the formation of resting structures in different Aspergilli [44,45,46]. In addition to their developmental impact, fungal oxylipins can modulate mycotoxin production and pathogenicity, for example, by stimulation of fungal lipid metabolite biosynthesis, which can influence plant host colonization [47]. Furthermore, oxylipins might mimic plant signal molecules in order to manipulate the host lipid metabolism and alter plant defense responses. The interplay between plant and fungal oxylipins can direct the outcome of the fungus-plant interaction [48].

Several filamentous fungi produce oxylipins instead of pheromones as signaling molecules to coordinate developmental responses. Perception of these intra- or extracellular signals can occur via mitogen-activated protein kinase (MAPK) pathways such as the pheromone response MAPK pathway. The pheromone response MAPK pathway was originally described in *Saccharomyces cerevisiae* for a-cell-α-cell fusion during mating where the core module consists of the Ste50 adaptor, the Ste11 MAP kinase kinase kinase (MAP3K), the Ste7 MAP kinase kinase (MAP2K), and the Fus3 MAP kinase (MAPK). A phosphorylation signal is sequentially transferred with the help of the scaffold protein Ste5 to regulate downstream targets such as the transcription factor Ste12 [49]. Components of this pheromone response MAPK core can also participate in other Ste5 independent MAPK pathways, such as the starvation-mediated filamentous growth pathway of yeast comprising the Ste50 adaptor, the MAP3K Ste11, and MAP2K Ste7 as well as the MAPK Kss1 [50]. The pheromone response MAPK pathway plays an important role in the pathogenicity of numerous filamentous fungi [51]. The *V. dahliae* pheromone response MAPK pathway includes orthologues to the yeast pathway components (Adapter: Vst50; MAP3K: Vst11; MAP2K: Mek2/Vst7; MAPK: Vmk1; Ste12-like transcription factor: Vph1) and is essential for pathogenicity [52,53,54,55,56]. Vmk1, Mek2, Vst11, and Vst50-deficient mutants display defects in microsclerotia development, whereas resting structure formation is unaffected in a *VPH1* deletion strain [52,53,54,55,56].

A cross-regulation of the pheromone response MAPK pathway and the ER-associated UPR pathway was described in *U. maydis*. Pathogenicity of the smut fungus depends on activation of the UPR after invasion of the plant surface [33,57]. When activated prior to entering the plant cell, the UPR inhibits filamentous growth and virulence by reduction of the pheromone response MAPK pathway activity through dephosphorylation of the MAPK Kpp2, which is mediated by the dual-specificity phosphatase Rok1 [18,58]. In this fungus, *rok1* deficient mutant strains displayed increased filamentation and a hyper-virulent phenotype with increased appressorium formation on maize plants.

In this study, we analyzed the impact of the UPR, the pheromone response MAPK pathway scaffold protein and the MAPK phosphatase Rok1 on the development and pathogenicity of the vascular plant pathogen *V. dahliae.* We show that a functional UPR contributes to growth and conidiation of *V. dahliae* and is required for successful colonization of host plants. The UPR regulator Hac1 is essential for the formation of dormant structures, which is critical for the ability of the fungus to persist in the soil for many years and to successfully re-establish the disease in the next season. The ER-associated oleate Δ12-fatty acid desaturase producing linoleic acid as precursor of oxylipin hormones primarily promotes fungal growth with only minor impact on the pathogenicity of *V. dahliae*. The pheromone response MAPK pathway promotes *V. dahliae* resting structure formation, growth, and virulence independently of the Ham5 protein described as a MAPK scaffold protein in other filamentous ascomycetes [59,60,61]. The *V. dahliae* dual-specificity MAPK phosphatase Rok1 promotes fungal growth, conidiation, and virulence and limits resting structures formation to an intermediate level. Overall, similar signaling component mutant phenotypes observed in this study suggest an interplay between the UPR and pheromone response MAPK signaling, which both affect fungal growth, resting structure development, and virulence in the vascular pathogen *V. dahliae*.

## 2. Materials and Methods

### 2.1. Bacterial and Fungal Cultivation Conditions

*Escherichia coli* DH5α cells (Invitrogen, Carlsbad, CA, USA) and *Agrobacterium tumefaciens* AGL1 [62] were used for plasmid preparation and *Verticillium* transformation, respectively. Bacteria were cultivated in lysogeny broth (LB) [63] or on solid LB supplemented with kanamycin (100 µg/mL, Sigma-Aldrich, St. Louis, MO, USA) or ampicillin (100 µg/mL, Carl Roth, Karlsruhe, Germany) at 37 °C for *E. coli* and 25–28 °C for *A. tumefaciens*, respectively.

*V. dahliae* strains were cultivated in liquid simulated xylem medium (SXM) modified from [64] as described in [65] to obtain conidiospores, or in liquid potato dextrose bouillon (PDM; Carl Roth, Karlsruhe, Germany) for mycelial growth at 25 °C shaking at 115–125 rpm. Conidia were harvested with sterile Miracloth (Calbiochem, Kenilworth, NJ, USA) and resuspended in sterile water. Conidiospore concentrations were determined using the Coulter Z2 Particle Count and Size Analyzer (Beckman Coulter, Brea, CA, USA) and the appropriate Coulter Isoton II Diluent. Spores were conserved as 25% glycerol stocks at −80 °C. Mycelium was harvested through Miracloth, rinsed with 0.96% NaCl solution, dried, and frozen in liquid nitrogen prior to isolation of genomic DNA, RNA or proteins.

*Verticillium* transformants were selected using solid potato dextrose medium (PDM, ‘Potato dextrose agar’, Carl Roth, Karlsruhe, Germany; plus 0.5% agar) supplemented with nourseothricin (72 µg/mL; clonNAT, Werner BioAgents, Jena, Germany), hygromycin B (50 µg/mL, InvivoGen, San Diego, CA, USA), or geneticin G418 (50 µg/mL, Sigma-Aldrich, St. Louis, MO, USA), respectively, and cefotaxime (300 µg/mL; FUJIFILM Wako chemicals, Neuss, Germany) for elimination of *A. tumefaciens*.

PDM, SXM, modified Czapek Dox medium (CDM) [66], CDM with 3% cellulose, 3% galactose, or 3% glucose as alternative carbon sources, CDM supplemented with 0.125% linoleic acid (LA), CDM supplemented with different stress inducing agents (0.00075% H_2_O_2_, 0.004% SDS, 0.5 M NaCl, 0.8 M Sorbitol, 1 µg/mL tunicamycin), and plant agar (25% shredded tomato plants, 2% agar) were used for analysis of fungal growth and ex planta phenotypes. Plates were incubated at 25 °C and growth was observed at indicated time points.

### 2.2. Verticillium Strain Construction

*V. dahliae* JR2 wildtype [67] was used for construction of all *Verticillium* strains used in this study (Table S1) by *Agrobacterium tumefaciens*-mediated transformation (ATMT) [68]. The plasmids pGreen2 [69] and pME4819 were used for construction of green fluorescent strains. Plasmids pME4975 and pME4976 were used to construct strains with RFP-labeled histone H2b for localization studies. Plasmids pPK2 [70], pKO2 [71], pME4564 [71], pME4815 [71], pME3857 [72], and pCOM [73] were used as backbones for plasmid constructions. To construct deletion and complementation cassettes (Table S3), the GeneArt Seamless Cloning and Assembly Kit (Thermo Fisher Scientific, Waltham, MA, USA) and the FastCloning protocol [74] were used. Further details on plasmid and strain constructions are given in Methods S1. Isolation of fungal genomic DNA and Southern hybridization were performed as described in [71]. Southern hybridizations of constructed strains are shown in Figures S1–S4. Primers are listed in Table S2 and plasmids in Table S3.

### 2.3. Quantification of Growth and Developmental Structures

Growth of *Verticillium* strains was quantified by spotting 5 × 10^4^ conidiospores on 30 mL solid media as indicated. Colony diameters were determined by measurement of two perpendicular diameters per colony and three plates per transformant and medium (*n* = 1). Two independent *HAC1^i^-HA* and *HAC1^u^-HA* transformants each were tested in comparison to a single *HAC1-C* transformant and wildtype and either one or two ∆*HAC1* transformants in three independent experiments. For quantification of growth 3, 6, and 9 d growth quantification of *ODE1* strains, two independent ∆*ODE1* and *ODE1-GFP* complementation transformants were tested in comparison to wildtype in two independent experiments. Three independent experiments for quantification of growth on plant agar were performed with a single ∆*ODE1* and *ODE1-GFP* complementation transformant each. Two independent transformants were tested per single deletion strain (∆*HAM5*, ∆*VMK1*, and ∆*MEK2*) in comparison to a single transformant each per double deletion strain (∆*HAM5*∆*VMK1* and ∆*HAM5*∆*MEK2*), complementation strain (*HAM5-C*, *VMK1-C*, and *MEK2-C*) and wildtype in three independent experiments each. Significances were calculated using two-tailed Student’s *t*-test.

Colony cross sections were analyzed with a binocular microscope SZX12-ILLB2-200 (Olympus, Tokyo, Japan) and microsclerotia were observed with an Axiolab light microscope (ZEISS, Oberkochen, Germany).

Melanization of colony centers was quantified by determination of the brightness factor using ImageJ software [75]. Nine days after spot inoculation of 5 × 10^4^ freshly harvested spores on 30 mL CDM with cellulose plates, top view pictures from colonies were taken after removal of aerial mycelium and the melanized area was measured from eight bit greyscale pictures. The brightness factor of the agar background was subtracted and means were set relative to wildtype. Three colonies per transformant were considered as one biological replicate (*n* = 1). Two independent transformants were tested per single deletion strain (∆*HAM5*, ∆*VMK1*, and ∆*MEK2*) in comparison to a single transformant each per double deletion strain (∆*HAM5*∆*VMK1* and ∆*HAM5*∆*MEK2*), complementation strain (*HAM5-C*, *VMK1-C*, and *MEK2-C*) and wildtype in three independent experiments each. Melanization of a single ∆*ROK1* and *ROK1-C* complementation strain each was tested in comparison to wildtype in three independent experiments. Significances were calculated using two-tailed Student’s *t*-test.

Quantification of conidiospores was performed 5 d after inoculation of freshly harvested conidia in liquid SXM to a concentration of 4 × 10^3^ conidiospores per mL and incubation at 25 °C under constant agitation at 120–135 rpm. Conidia were filtered through Miracloth, diluted in equal volumes of sterile water, and counted. Concentrations were determined in triplicates per transformant (*n* = 1). Two independent ∆*HAC1* transformants were tested in comparison to a single *HAC1-C* transformant and wildtype in four independent experiments. A single ∆*ROK1* and *ROK1-C* complementation transformant each was tested in comparison to wildtype in three independent experiments.

Conidiospore numbers were normalized to wildtype. Error bars indicate the standard deviations. Significances were calculated using two-tailed Student’s *t*-test.

### 2.4. Protein Extraction and Western Hybridization

For detection of Hac1-HA or Ode1-GFP proteins, 1 × 10^6^ freshly harvested spores were inoculated in 50 mL liquid PDM and incubated for 4 d at 25 °C shaking. For detection of phosphorylated Vmk1 proteins, 1 × 10^7^ freshly harvested spores were inoculated in 50 mL liquid PDM and incubated for 3 d at 25 °C shaking. Mycelium was transferred to liquid CDM with glucose as carbon source either with or without supplemented tunicamycin (1 µg/mL) for 1 d at 25 °C shaking. Protein extracts were obtained from mycelial powder mixed with B* buffer (300 mM NaCl, 100 mM Tris-HCl pH 7.5, 10% glycerol, 2 mM EDTA, 0.02% NP-4O), 2 mM DTT, and cOmplete Protease inhibitor cocktail mix (Roche, Basel, Switzerland). For detection of phosphorylated Vmk1, additionally PhosSTOP phosphatase inhibitor cocktail (Roche, Basel, Switzerland) and phosphatase inhibitor mix (1 mM NaF, 0.5 M sodium-orthovanadate, 8 mM β-glycerolphosphate disodium pentahydrate) were supplemented. The cell debris was sedimented by 30 min centrifugation at 13,000 rpm and 4 °C. Protein concentration was determined by Bradford-based Roti-Quant assay (Carl Roth, Karlsruhe, Germany) using the Infinite M200 microplate reader operated with Magellan software (Tecan Trading, Männedorf, Switzerland). 80 µg protein extracts were loaded on 12% SDS gels and transferred onto nitrocellulose membranes (Amersham Protran 0.45 μm, GE Healthcare, Chicago, IL, USA). Ponceau S (0.2% Ponceau S, 3% TCA) staining was used as loading control. Membranes were incubated with monoclonal mouse α-HA (H9658, Sigma-Aldrich, St. Louis, MO, USA), monoclonal mouse α-GFP IgG (sc-9996, Santa Cruz Biotechnology, Dallas, TX, USA) or monoclonal rabbit phospho-p44/42 MAPK (Erk1/2) (Thr202/Tyr204) antibody (197G2, Cell Signalling Technology, Danvers, MA, USA). As secondary antibody, polyclonal goat α-mouse IgG (115-035-003, Jackson Immuno Research, West Grove, PA, USA) or polyclonal goat α-rabbit IgG (G-21234, Invitrogen, Carlsbad, CA, USA) was used, respectively. Horseradish peroxidase (HRP) substrate luminol-based chemiluminescence was exploited for detection of proteins. Signals were visualized using Amersham Hyperfilm ECL film (GE Healthcare, Chicago, IL, USA), which was developed with the Optimax (Protec, Oberstenfeld, Germany) film processor. For quantification of signal intensity, signals were visualized with the Fusion SL chemiluminescence detector (Peqlab Biotechnology, Erlangen, Germany), operated with the corresponding software Fusion 15.18 (Vilber Lourmat, Collégien, France). Single Δ*ROK1* and *ROK1-C* transformants were compared to wildtype from three independent biological replicates.

### 2.5. Isolation of RNA, cDNA Synthesis, and Quantification of Gene Expressions

For isolation of RNA 1 × 10^6^ spores were inoculated in 50 mL SXM and incubated for 3 d at 25 °C under constant agitation. RNA was purified using the Direct-zol RNA MiniPrep Kit (Zymo Research, Irvine, CA, USA) according to the manufacturer’s instructions. RNA integrity was tested by gel electrophoresis. Reverse transcription to cDNA was performed using the QuantiTect Reverse Transcription Kit (Qiagen, Hilden, Germany) according to the manufacturer’s protocol. Contaminations by gDNA were checked as described in [66] prior to quantification of gene expressions. Transcription levels were analyzed in triplicates (*n* = 1) using a CFX Connect Real Time System cycler (Biorad, Hercules, CA, USA) with Mesa Green qPCR MasterMix Plus for SYBR Assay (Eurogentec, Liège, Belgium). Primers JST325/326 (*BIP1*), JST290/JST291 (*HAC1* variants), SZ9/SZ10 (*H2A*), and SZ11/SZ12 (*EIF2B*) were used. Expression levels of *BIP1* and *HAC1* were quantified relative to the reference genes histone *H2A* (*VDAG_JR2_Chr4g01430a*) and *EIF2B (VDAG_JR2_Chr4g00410a*) by qRT PCR using the ΔΔCT method [76]. Two independent transformants of *HAC1^i−^HA* and *HAC1^u−^HA* were compared to a single *HAC1* deletion, a *HAC1-C* transformant and wildtype in three independent experiments. Significances were calculated using two-tailed Student’s *t*-test.

### 2.6. Confocal Microscopy

Localization of Ode1-GFP was analyzed by confocal fluorescence microscopy. Circa 5 × 10^4^–1 × 10^5^ freshly harvested spores were used for inoculation of µ-slide 8 well microscopy chambers (ibidi) with 300 µL liquid PDM per well and incubated at 25 °C for the indicated time. Morphology of hyphae and subcellular localization were examined using a Plan-Neofluar 100×/1.4 oil objective (Zeiss, Oberkochen, Germany; GFP: 300 ms exposure time; RFP: 800 ms exposure time).

### 2.7. Arabidopsis thaliana Root Colonization Assays

The root colonization assays were performed as described in [66]. Fluorescence microscopy of the roots was conducted at the indicated time points. Overview pictures (20× objective (Zeiss, Oberkochen, Germany)) and close up pictures (63× objective (Zeiss, Oberkochen, Germany)) were taken (GFP: 300 ms exposure time; RFP: 800 ms exposure time). At least two independent plants were examined per experiment, fungal strain, and time point.

### 2.8. Tomato Plant Infection Assays

Tomato plant infection experiments were performed as described in [66]. The disease symptoms were scored at 21 d post infection according to the following disease rating criteria: The fresh weight excluding the roots, the length of the longest leaf, and the height of the vegetation point per plant were determined and transformed into a score ranging from 1 to 5 relative to the mean values determined for the uninfected Mock plants (70–100% (Mock) = 1; 60–69% (Mock) = 2; 40–59% (Mock) = 3; <40% (Mock) = 4; dead plant = 5). The mean of the scores for each parameter (height/length of the longest leaf/weight) determined the disease score per plant, which ranges from 1 to 5. Plants were categorized according to the disease score as follows: 1–1.99 = healthy; 2–2.99 = weak symptoms; 3–3.99 = strong symptoms; 4–4.99 = very strong symptoms; 5 = dead plant. The number of plants categorized in the disease scores 1 to 5 relative to the total number of tested plants was visualized in stack diagrams. Plants inoculated with spores of two independent transformants with the same genotype are shown in one bar. Two independent ∆*HAC1* transformants were tested in comparison to wildtype and a single *HAC1-C* transformant, each in two independent plant infection experiments. Two independent ∆*ODE1* and *ODE1-GFP* transformants were tested in comparison to wildtype (five independent tests each) in three independent plant infection experiments. Two independent ∆*HAM5* transformants were tested in three independent experiments each in comparison to a single transformant each per double deletion strain (∆*HAM5*∆*VMK1*, ∆*HAM5*∆*MEK2*), complementation strain (*VMK1-C*, *MEK2-C*) and wildtype as well as a single or two independent ∆*VMK1* (four independent tests) and ∆*MEK2* (five independent tests) single deletion strains. A ∆*ROK1* and a corresponding complementation strain *ROK1-C* were compared to wildtype in five independent tests and in three plant infection experiments.

The discoloration of the hypocotyl was determined by observation of cross sections. To test for fungal outgrowth from stems of infected plants, harvested stems were surface sterilized (70% ethanol for 8 min, 6% sodium hypochloride solution for 8 min, and two washing steps with distilled sterile water). The stem ends were removed, and stem slices were incubated on PDM plates supplemented with chloramphenicol (100 µg/mL; AppliChem, Darmstadt, Germany) for 7 d at 25 °C. Fungal outgrowth was documented.

### 2.9. Sequence Analyses

BLAST searches were performed using online databases National Center for Biotechnology Information (NCBI; [77]) and Ensembl Fungi [78]. *Verticillium* gene predictions and sequences as well as accession numbers were obtained from Ensembl Fungi. Prediction of mRNA secondary structures was performed using the web server RNAfold [79]. Protein domain predictions were analyzed using InterPro website (http://www.ebi.ac.uk/Tools/pfa/iprscan (accessed on 14 April 2021) [80]). The online databases cNLS mapper (http://nls-mapper.iab.keio.ac.jp/cgi-bin/NLS_Mapper_form.cgi (accessed on 14 April 2021 [81], and DeepLoc-1.0; (http://www.cbs.dtu.dk/services/DeepLoc/index.php (accessed on 14 April 2021) [82]) were used for prediction of nuclear localization signals. ClustalW [83] or Muscle [84] algorithms were used for multiple alignment of protein sequences with MEGA6.0 [85] software. Phylogenetic analysis was conducted using Maximum likelihood tree method in MEGA6.0 software [85].

## 3. Results

### 3.1. The Regulator of the Endoplasmic Reticulum-Associated Unfolded Protein Response Pathway Hac1 Supports Fungal Growth and Is Essential for Resting Structure Development in V. dahliae

The UPR monitors secretion capacity and protein folding in the ER. This is critical for fungal differentiation processes and colonization-related adaptation. Different environmental signals are perceived at the cell membrane, transduced to the nucleus, and change ER-mediated secretion as appropriate cellular response to the external trigger.

**Figure 1:**
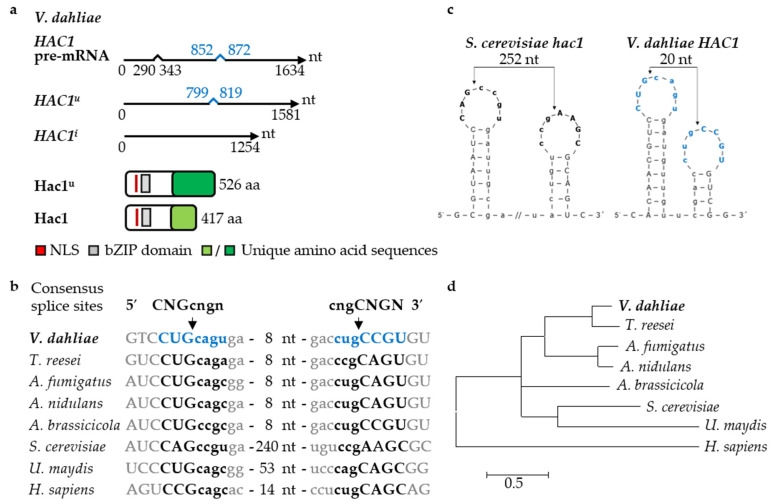
The *Verticillium dahliae* unfolded protein response regulator Hac1. (**a**) *V. dahliae HAC1* mRNA and protein variants. The unspliced *HAC1* pre-mRNA (1634 nt) contains a conventional (53 nt, black) and an unconventional (20 nt, blue) intron. Two *HAC1* mRNA variants result from splicing of either the conventional intron, resulting in *HAC1^u^* mRNA (1581 nt) encoding the potential 526 aa Hac1^u^ protein or additional splicing of an unconventional intron, resulting in the shorter induced *HAC1^i^* mRNA (1254 nt) coding for the 417 aa Hac1 protein. Both proteins possess identical N-termini (268 aa) with basic leucine zipper domain (bZIP, grey, PS50217; 107–164 aa) and a nuclear localization signal (NLS, red, 94–105 aa), whereas Hac1^u^ and Hac1 C-termini are unique (dark vs. light green). (**b**) Unconventionally spliced introns of UPR regulators show high conservation. Sequences of 5′- and 3′-splice sequences of *V. dahliae HAC1*, *Trichoderma reesei hac1* (M419DRAFT_128619), *Aspergillus fumigatus hacA* (XM_743634), *Aspergillus nidulans hacA* (AN9397), *Alternaria brassicicola HacA* [31], *Saccharomyces cerevisiae hac1* (NC_001138.5), *Ustilago maydis cib1* (UMAG_11782), and *Homo sapiens XBP1* (NM_005080.3) were aligned. (CNG’CNGN = consensus sequence [86], arrows = cleavage sites, lowercase characters = intron sequences, capital letters = splice sequences, numbers of nucleotides are given for those not shown). (**c**) Predicted twin stem-loop secondary structures of 5′- and 3′-splice sequences of unconventional *V. dahliae* and *S. cerevisiae HAC1* introns. (Arrows = cleavage sites, bold characters = splice sequences, lowercase characters = introns, // = discontinuation of intron sequence) (**d**) Phylogenetic tree derived of Hac1-like proteins. *V. dahliae* Hac1 (Figure S6), *T. reesei* HACI^i^ (XP_006964054.1), *A. fumigatus* HacA^i^ (ACJ61678.1), *A. nidulans* HacA^i^ (Q8TFU8.2), *A. brassicicola* AbHacA [31], *S. cerevisiae* HAC1^i^ (NP_116622.1), *U. maydis* cib1^s^ (XP_011390112.1) and *H. sapiens* XBP1 (NP_001073007.1) sequences were used (Muscle algorithm, scale bar = average number of amino acid substitutions per site).

A Hac1-like UPR transcription factor was predicted in *V. dahliae* by BLASTp search. The *HAC1* gene carries a 53 nucleotide (nt) conventional intron and an additional non-conventional 20 nt intron. Splicing of the conventional intron results in a *HAC1^u^* transcript of 1581 nt encoding a protein with 526 amino acids (aa). Additional splicing of the non-conventional intron results in *HAC1^i^* with an altered reading frame of 1254 nt coding for the 417 aa Hac1 protein. Expression of the larger *HAC1^u^* as well as the smaller *HAC1^i^* splice variants was verified by characterizing amplified cDNA sequences. Only *HAC1^i^* transcripts encoding Hac1 were found in the presence of ER stress mediated by dithiothreitol (DTT; induced conditions), whereas without DTT (uninduced conditions) both, *HAC1^u^* and smaller *HAC1^i^*, were identified (Figure 1a–c, Figures S5 and S6).

The length of 20 nt for the unconventionally spliced intron of *V. dahliae HAC1* is similar to other filamentous ascomycetes (20–26 nt), but smaller than in the basidiomycete *U. maydis* (65 nt) or in budding yeast (252 nt) (Figure 1b). Unconventional splicing of mRNAs of *HAC1* homologs requires the cytosolic endoribonuclease domain of the ER membrane-resident sensor Ire1. This domain recognizes the conserved consensus splice site 5′-CNG’CNGN-3′ [86]. 5′- and 3′-intron-exon-borders of the 20 nt intron from *V. dahliae HAC1* are conserved (Figure 1b). Similar to Hac1 splice sites of different organisms, the consensus splice site of the unconventional *V. dahliae HAC1* intron was predicted to form a characteristic twin stem-loop secondary structure (Figure 1c).

The two *HAC1* splice variants encode proteins with identical N- but different C-terminal regions. The shared N-terminal 268 aa region includes the nuclear localization signal and the bZIP domain (Figure 1a). The remaining C-termini of the deduced larger 526 aa Hac1^u^ protein of 58 kDa and the smaller 417 aa Hac1 protein of 44 kDa are unique (Figure 1a, the amino acid sequence of Hac1 is shown in Figure S6). A phylogenetic analysis revealed similarities between the *V. dahliae* Hac1 protein encoded by the unconventionally spliced mRNA *HAC1^i^* to described UPR regulatory proteins in other fungi and to human XBP1 (X-box binding protein) (Figure 1d). Proteins from *V. dahliae* and *T. reesei* cluster in one subclade. Proteins with less similarity to *V. dahliae* Hac1 are corresponding proteins of the *S. cerevisiae* and *U. maydis* cluster.

**Figure 2:**
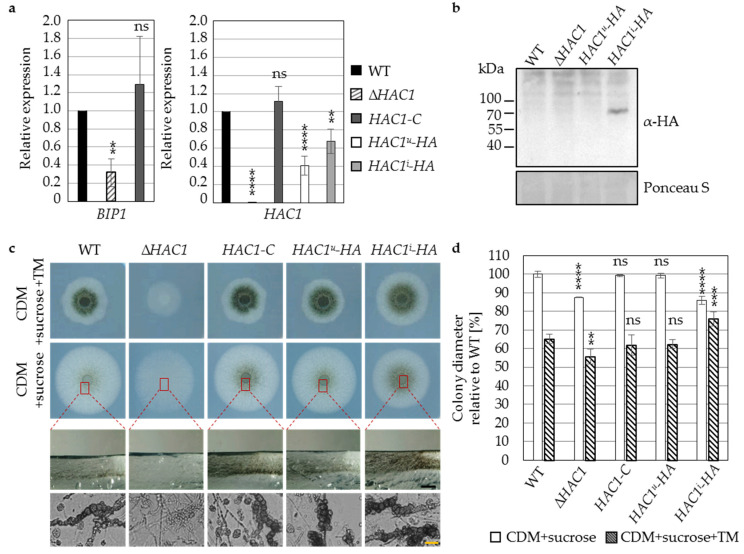
*Verticillium dahliae HAC1* supports growth and is essential for microsclerotia formation. *V. dahliae* wildtype (WT), *HAC1* deletion (∆*HAC1*), and complementation (*HAC1-C*) strains, as well as strains expressing ectopically integrated *HAC1* mRNA splice variants fused to HA at the 3′-end in the ∆*HAC1* strain (*HAC1^u^-HA*; *HAC1^i^-HA*) were compared. (**a**) Quantification of *BIP1* (left) and *HAC1* (right) gene expression levels. Primers targeting *BIP1* or both *HAC1* mRNA splice variants were used. Bars represent mean values normalized to wildtype with *H2A* and *EIF2B* as references with standard deviations. (Significant differences to wildtype: ** *p* < 0.01; **** *p* = 0; ns = non-significant; *n* ≥ 3). *HAC1^u^-HA* and *HAC1^i^-HA* show reduced *HAC1* gene expression levels. (**b**) Immunoblot of Hac1 proteins in *HAC1^u^-HA* and *HAC1^i^-HA*. HA-specific antibody and Ponceau S staining as loading control were used. For *HAC1^i^-HA* producing unconventionally spliced *HAC1* mRNA only, a signal is visible at ~70 kDa for tagged Hac1 instead of the predicted 46 kDa. In both strains no specific band was observed for the predicted Hac1^u^-HA protein (59 kDa). (**c**) Microsclerotia formation ex planta. Except ∆*HAC1*, all strains form wildtype-like microsclerotia 10 d after spot inoculation on CDM with sucrose with or without tunicamycin (TM = 1 µg/mL). For ∆*HAC1* neither melanized nor unmelanized microsclerotia are visible in cross sections of the colony centers (red boxes/dashed lines) or microscopy of fungal material (bottom). *HAC1-C* and *HAC1^i^-HA* strains produce increased levels (Black scale bar = 1 mm, yellow scale bar = 20 µm). (**d**) Quantification of vegetative growth 10 d after spot inoculation. ∆*HAC1* displays reduced growth. *HAC1-C* and *HAC1^u^-HA* display wildtype-like growth, whereas growth of *HAC1^i^-HA* is decreased under non-stress conditions and increased upon supplementation of TM. Mean values of three independent experiments relative to wildtype on CDM with sucrose and standard deviations are shown. (Significant differences to wildtype on respective media: ** *p* < 0.01; *** *p* < 0.001 **** *p* = 0, ns = non-significant, *n* ≥ 3).

A deletion strain was constructed to analyze the role of *HAC1* in the development and virulence of *V. dahliae.* This mutant strain was used for ectopic reintegration of the entire *HAC1* gene (*HAC1-C*). Hac1-dependent gene expression levels of the ER chaperone Bip1, a conserved UPR target gene in yeast and filamentous fungi [18], were determined by quantitative reverse transcription PCR. *V. dahliae BIP1* (*VDAG_JR2_Chr3g10940a*) was identified as homolog of the *Aspergillus nidulans* BipA encoding gene with high amino acid sequence identity of ~76% for the 666 aa protein. Expression of *BIP1* was significantly reduced (~70% lower) in the *HAC1* deletion strain grown in liquid simulated xylem medium for three days (Figure 2a), suggesting that the ER-resident chaperone Bip1 encoding gene is a target of the UPR transcription factor Hac1 in *V. dahliae*.

Next, strains were constructed containing either one of the two mRNA *HAC1* variants (Figure 1a) fused to a hemagglutinin (*HA*) tag at the 3′-end and expressed under control of the native promoter and terminator in the *HAC1* deletion background. Expression levels of induced and uninduced *HAC1* mRNA variants were examined with the same primer. *HAC1^i^-HA* (~67%) and *HAC1^u^-HA* (40%) strains harboring either one of two *HAC1* splice variants displayed decreased expression compared to *HAC1* gene expression levels of wildtype or complementation strain (Figure 2a). This altered transcriptional activity of the construct might result from different genomic contexts after ectopic integration.

To investigate whether both *HAC1* mRNA variants are translated into Hac1^u^-HA and Hac1-HA fusion proteins, immunoblot analysis was conducted. A signal at ~70 kDa instead of the predicted 46 kDa was obtained for *HAC1^i^*-*HA*, expressing the unconventionally spliced *HAC1* mRNA variant, whereas no signal was detected for the *HAC1^u^-HA* strain containing the mRNA variant where only the conventional intron was spliced (Figure 2b). Band shifts might indicate posttranslational modifications or are caused by stable secondary structures of the protein, resistant to denaturation procedures.

Alterations in growth and development of the constructed strains ex planta were compared to wildtype. ∆*HAC1* colonies produce less aerial mycelium and appear more transparent. Under non-stress conditions reduced growth was observed for ∆*HAC1* (12%) and *HAC1^i^-HA* (14%) in comparison to the wildtype strain (Figure 2c,d). Under tunicamycin-induced ER stress conditions growth of ∆*HAC1* was decreased (9% smaller), whereas *HAC1^i^-HA* displayed relatively increased colony diameters (11% bigger) compared to wildtype. The expression of the ectopically integrated *HAC1* gene in *HAC1-C* or the *HAC1* gene lacking the conventional intron in *HAC1^u^-HA* in the deletion background resulted in wildtype-like growth under stress and non-stress conditions. This suggests that the presence of the unconventionally spliced *HAC1* mRNA in the *HAC1^i^-HA* strain enables a more efficient response to ER stress.

For ∆*HAC1* no melanization of the colony centers was observed during growth on any tested medium. Cross sections and microscopy of fungal material from ∆*HAC1* colonies grown on different media revealed the absence of microsclerotia, whereas ectopic integration resulted in increased resting structure occurrence for the *HAC1-C* and *HAC1^i^-HA* strains, as exemplified for minimal medium with sucrose as carbon source (Figure 2c). Wildtype-like microsclerotia amounts were observed in the *HAC1^u^-HA* strain. The absence of microsclerotia in ∆*HAC1* and vice versa increased melanization in *HAC1^i^-HA* corroborate a regulatory function of Hac1 in formation of resting structures.

### 3.2. Virulence of V. dahliae Depends on the Unfolded Protein Response Transcription Factor Hac1

**Figure 3:**
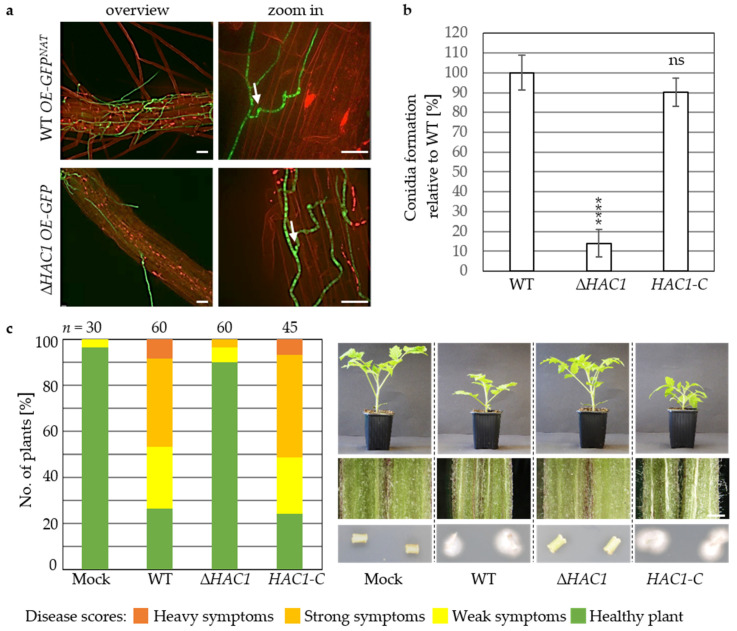
*Verticillium dahliae HAC1* is required for root colonization, conidiation, and induction of disease symptoms in tomato. *V. dahliae* wildtype (WT), *HAC1* deletion (∆*HAC1*), and complementation (*HAC1-C*) strains, as well as wildtype and *HAC1* deletion strain constitutively expressing ectopic GFP (WT *OE-GFP^NAT^*, Δ*HAC1 OE-GFP*) were compared. (**a**) *V. dahliae* colonization of *Arabidopsis thaliana* roots. Fluorescence confocal microscopy was performed 7 d post inoculation of roots with the same numbers of spores from wildtype *WT OE-GFP^NAT^* or ∆*HAC1 OE-GFP* with four plants per strain in two independent experiments. ∆*HAC1 OE-GFP* propagation on the root surface is reduced, but penetration of the root (white arrows) with subsequent colonization of the root cortex was observed (Scale bar = 20 µm). (**b**) Quantification of conidiation in ∆*HAC1*. ∆*HAC1* shows reduced conidiation 5 d post inoculation of liquid simulated xylem medium in comparison to wildtype and *HAC1-C*. Mean values of four independent experiments relative to wildtype with standard deviations are shown. (Significant differences to wildtype: **** *p* = 0, ns = non-significant, *n* ≥ 4). (**c**) Pathogenicity test of Δ*HAC1* mutant compared to wildtype towards *Solanum lycopersicum*. Representative tomato plants (right, top) and hypocotyl dissections (right, middle) 21 d after root dipping into distilled water control (Mock) or same numbers of spores obtained from different strains are shown (Scale bar = 1 mm). Relative amount of plants with certain disease scores from two independent experiments are displayed in the stack diagram (*n* = total number of evaluated plants). Δ*HAC1*-treated plants display disease symptoms in only few plants, mock-like hypocotyl coloration and no fungal outgrowth from surface sterilized stem sections after 7 d (bottom).

It is yet unknown whether the UPR regulator Hac1 is required for fungal pathogens that colonize plant roots. For monitoring root colonization, *GFP* under control of the *gpdA* promoter was ectopically integrated into the *HAC1* deletion strain. Colonization of *Arabidopsis thaliana* roots by the resulting ∆*HAC1 OE-GFP* strain was studied seven days after inoculation with the same numbers of spores in comparison to the wildtype carrying the same construct (WT *OE-GFP^NAT^*). Overall, less hyphae were present on the root surface for the Δ*HAC1 OE-GFP* strain (Figure 3a). The formation of swollen hyphae and a change in growth direction indicate penetration sites. These features were observed in the absence of *HAC1* similarly to wildtype, as was hyphal growth after invasion of the outer root layer. Therefore, *V. dahliae* Hac1 is not required for penetration of the root and the mutant is not blocked directly after invasion of the root cortex, but Hac1 supports the first contact with the host, which is the initial colonization of the root surface.

*V. dahliae* forms conidiospores within the plant vascular system for spreading and systemic colonization. Conidiospore numbers were significantly reduced in the *HAC1* deletion strain with about 14% formed conidia relative to wildtype after five days in liquid simulated xylem medium, whereas for *HAC1-C* wildtype-like numbers were obtained (Figure 3b). This suggests a further role for Hac1 in subsequent steps of plant colonization, and this was investigated by tomato infection experiments. ∆*HAC1*-treated plants displayed less severe disease symptoms than wildtype-treated plants 21 days after inoculation with conidiospores, resulting in approximately 90% healthy plants (Figure 3c). No heavy symptoms were determined in ∆*HAC1*-inoculated plants and no hypocotyl discolorations were observed for any of these plants. In addition, no fungal outgrowth was observed for ∆*HAC1* from stems of treated plants (Figure 3c). The greatly reduced virulence of the ∆*HAC1* strain in planta was complemented in the *HAC1-C* complementation strain. These results support the conclusion that regulation of UPR target genes, monitoring of the secretion capacity, and protein folding in the ER by Hac1 are required for the initial colonization of the host root surface and sporulation as prerequisite for fungal propagation within the plant. Hac1 is therefore required for induction of severe disease symptoms in tomato.

### 3.3. The Endoplasmic Reticulum-Associated V. dahliae Oleate ∆12-Fatty Acid Desaturase Ode1 Promotes Fungal Differentiation with Only a Minor Impact on Virulence

Secretion of lipid metabolites relies on the UPR control of ER-mediated secretion. To coordinate their development, several filamentous fungi produce oxylipins as signaling molecules, which are connected to host–fungus communication [44,47,87,88,89,90,91]. We examined functions of *V. dahliae* oxylipins as fungal signals for development and virulence and focused on an oleate ∆12-fatty acid desaturase catalyzing the oxidation of oleic acid to linoleic acid, the major precursor of fungal oxylipins, which has not yet been examined in plant pathogens.

**Figure 4:**
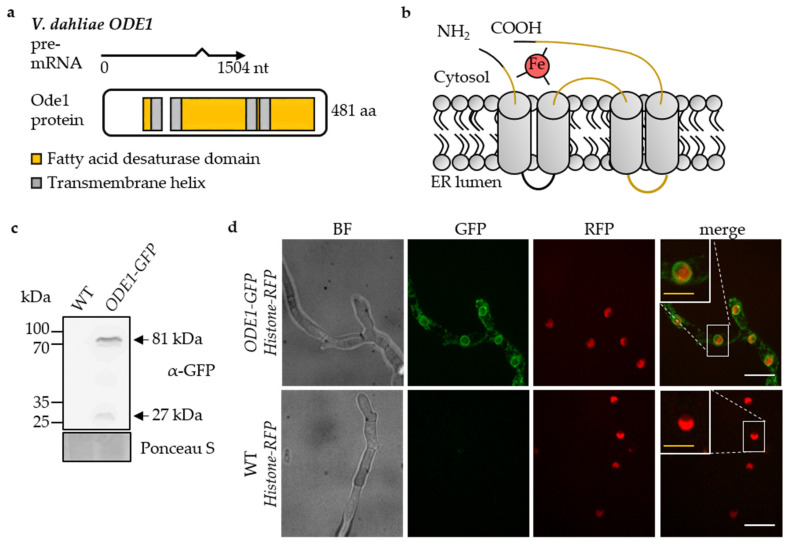
*Verticillium dahliae* Ode1 is localized to nuclear membranes. The *ODE1-GFP* strain harbors a functional fusion at the endogenous locus controlled by the native promoter. This strain with or without RFP-tagged histone H2b, and wildtype constitutively expressing RFP-tagged histone H2b (WT *Histone-RFP*) were compared. (**a**) *V. dahliae* oleate ∆12-fatty acid desaturase encoding *ODE1* intron-exon boundaries (1504 bp) were confirmed by PCR amplification from wildtype cDNA and sequencing. The deduced Ode1 protein (481 aa) contains two fatty acid desaturase domains (FAD; yellow; 77–112 aa, IPR021863; 138–424 aa; IPR005804) and four putative transmembrane helices (grey: 105–124 aa, 136–157 aa, 300–319 aa, 331–350 aa; Phobius). (**b**) Scheme of the predicted Ode1 protein structure. Ode1 is a transmembrane protein. N- and C-termini of Ode1 are directed to the cytosol. The catalytic center is formed by an iron atom (Fe) and the FAD domains (yellow). (**c**) Detection of Ode1-GFP (81 kDa) in immunoblot analysis using GFP-specific antibody. Ponceau S staining served as loading control. (**d**) Ode1-GFP is localized to membranes surrounding red nuclei in the *ODE1-GFP Histone-RFP* strain. Fluorescence microscopy 16 h post inoculation (yellow scale bar = 20 µm, white scale bar = 10 µm).

*V. dahliae ODE1* was identified as the homolog to the *A. nidulans* oleate ∆12-fatty acid desaturase *odeA* by reciprocal BLAST search of the amino acid sequences with 66% aa sequence identity of the deduced proteins. The 481 aa Ode1 protein with a predicted molecular weight of 54 kDa contains two N- and C-terminal histidine clusters described as fatty acid desaturase (FAD) domains (Figure 4a). Together with iron atoms provided by the membrane-bound donor cytochrome b_5_ the FAD domains form the cytosolic catalytic center of ∆12-fatty acid desaturases [92]. *V. dahliae* Ode1 was predicted as a transmembrane protein with cytosolic N- and C-termini, four hydrophobic transmembrane helices, and two short non-cytosolic regions (Figure 4b).

A *V. dahliae ODE1* deletion mutant and a complementation strain harboring *ODE1* with a 3′-*GFP*-tag at the endogenous locus under control of the native promoter were constructed. The predicted molecular weight for Ode1 fused to GFP (81 kDa) was confirmed by immunoblot analysis (Figure 4c). Fluorescence microscopy of young hyphae revealed that the Ode1-GFP protein is primarily localized to ER membranes surrounding the nucleus and to a minor extend to plasma membranes resembling perinuclear and cortical ER structures (Figure 4d).

**Figure 5:**
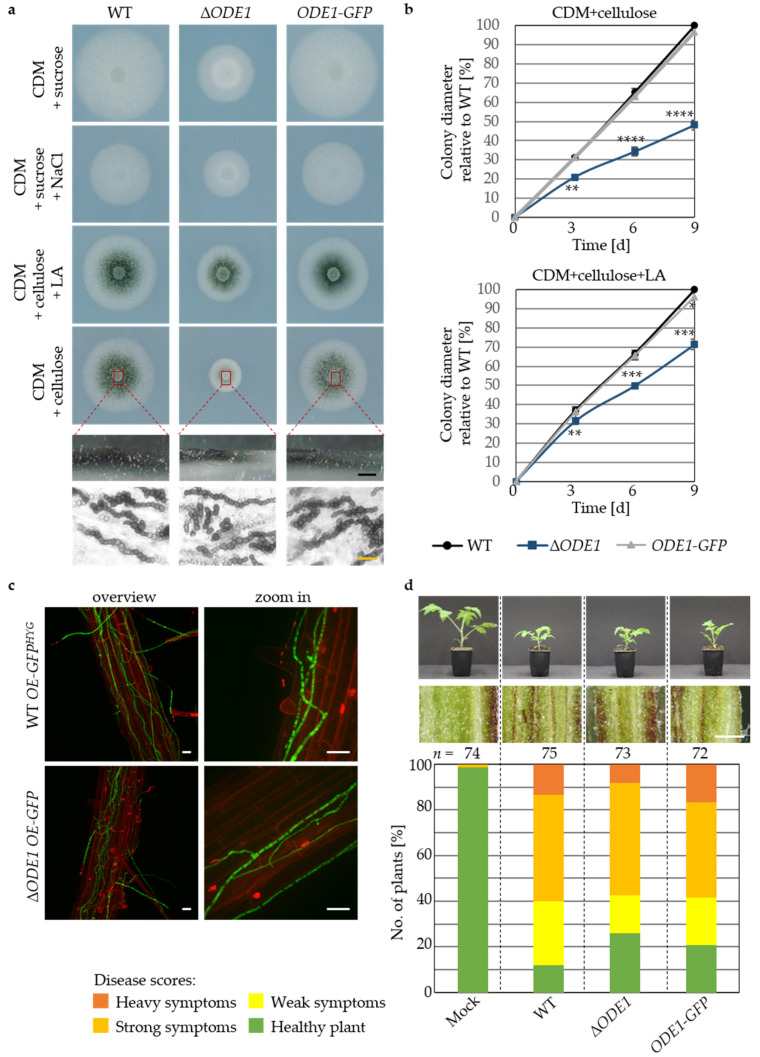
*Verticillium dahliae ODE1* contributes primarily to vegetative growth with only minor effects on plant disease symptoms. The *V. dahliae ODE1* deletion strain (∆*ODE1*), wildtype (WT), and the complementation strain harboring *ODE1-GFP* at the endogenous locus under control of the native promoter as well as wildtype and *ODE1* deletion strain constitutively expressing ectopic GFP (WT *OE-GFP^HYG^*, Δ*ODE1 OE-GFP*) were compared. (**a**) Vegetative growth and microsclerotia formation ex planta 9 d after spot inoculation on CDM supplemented with NaCl (0.5 M), CDM with either sucrose or cellulose, and CDM with cellulose supplemented with linoleic acid (LA, 0.125 mg/mL) are shown. Δ*ODE1* forms smaller colonies on all tested media. Cross sections of colonies grown on CDM with cellulose (red boxes/dashed lines) and microscopy of fungal material (bottom) display wildtype-like microsclerotia formation of Δ*ODE1* (black scale bar = 1 mm, yellow scale bar = 20 µm). (**b**) Growth quantification 3, 6, and 9 d after spot inoculation on CDM with cellulose either with or without supplemented linoleic acid (LA, 0.125 mg/mL). Δ*ODE1* displays about 50% decreased growth 9 d after spot inoculation on CDM with cellulose. LA supplementation partially complements the growth defect. Mean values relative to wildtype and standard deviations are shown (significant differences to wildtype on respective media: ** *p* < 0.01; *** *p* < 0.001; **** *p* = 0; *n* ≥ 3). (**c**) *V. dahliae* colonization of *Arabidopsis thaliana* roots. Fluorescence confocal microscopy was performed 5 d post inoculation of roots with the same numbers of spores from WT *OE-GFP^HYG^* or ∆*ODE1 OE-GFP* with four plants per strain in two independent experiments. ∆*ODE1 OE-GFP* propagation on the root surface is comparable to wildtype (Scale bars = 20 µm). (**d**) Pathogenicity test of *ODE1* mutant strains towards *Solanum lycopersicum*. Representative plants and hypocotyl dissections 21 d after root dipping into distilled water control (Mock) or same number of spores obtained from different strains are shown (Scale bar = 1 mm). Relative amount of plants with certain disease scores from three independent experiments are displayed in the stack diagram (*n* = total number of evaluated plants). Δ*ODE1* infection results in only a minor decrease in disease symptom induction.

The impact of the *V. dahliae* oleate ∆12-fatty acid desaturase Ode1 on fungal growth and differentiation was examined under different physiological and membrane stress inducing conditions. Vegetative growth of the ∆*ODE1* strain was significantly decreased relative to wildtype with or without stressors (Figure 5a,b).

The most severe decrease in vegetative growth was assessed on medium containing cellulose, with a ~50% reduction of the colony diameter after nine days (Figure 5a,b). The defect in growth of ∆*ODE1* was partially compensated by supplementation of media with linoleic acid, resulting in a relative colony diameter of about 70% (Figure 5a,b). Reduced melanization correlated with the decrease in growth of colonies formed by ∆*ODE1* on cellulose medium. Colony cross sections and microscopy of fungal material from colony centers revealed the formation of wildtype-like microsclerotia with regard to size, shape, and melanization by the ∆*ODE1* strain, suggesting that the reduced melanization depends on a reduced resting structure amount instead of reduced melanization of single microsclerotia. The phenotypes of the deletion strains could be restored to wildtype levels by *ODE1-GFP* complementation.

The impact of *ODE1* on propagation of *V. dahliae* on plant roots was examined by root dipping of *A. thaliana* plants into a spore solution of ∆*ODE1* and wildtype strains expressing ectopically integrated *GFP* for monitoring. Wildtype-like root colonization was observed for the *V. dahliae ODE1* deletion strain (Figure 5c). The impact of *V. dahliae* Ode1 on further colonization and resulting disease symptom induction was analyzed by tomato infection experiments. Tomato plants inoculated with ∆*ODE1* spores showed only slightly reduced numbers of plants with disease symptoms after 21 days compared to wildtype infected plants and no alterations in stunting or hypocotyl discolorations (Figure 5d). This suggests a minor contribution of the single gene *ODE1* to virulence of *V. dahliae*. Therefore, we tested if the *ODE1* deletion strain was able to compensate the absence of linoleic acid and its products using plant-derived linoleic acid. A partial compensation of the ∆*ODE1* growth defect (90%) was observed on plant agar prepared from shredded tomato plants (Figure S7). We conclude that *V. dahliae* Ode1 has a strong impact on fungal growth, which can be compensated by linoleic acid or its derived substances provided by the plant. Consequently, the ER-associated enzyme Ode1 has minor contribution to host plant colonization.

### 3.4. V. dahliae Development and Plant Disease Symptom Induction Require Pheromone Response MAP Kinase Activities Independently from the Ham5 Scaffold

**Figure 6:**
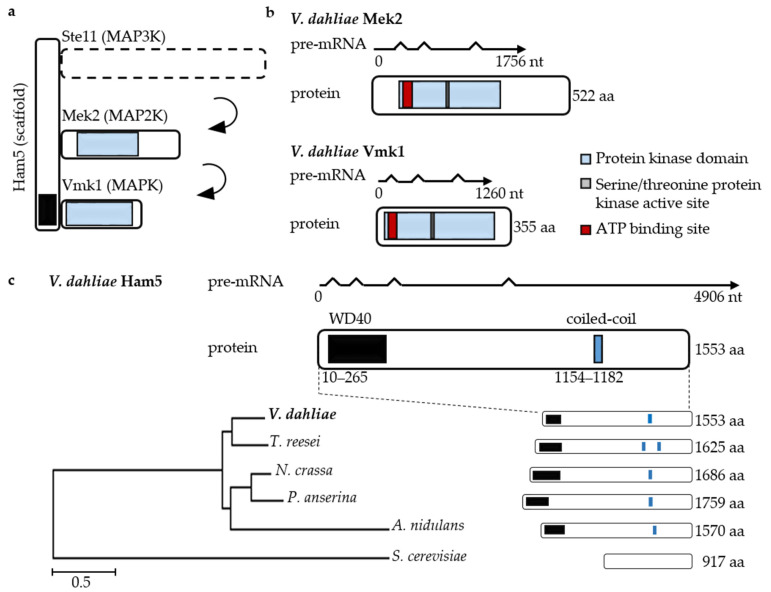
Pheromone response mitogen-activated protein (MAP) kinase module of *Verticillium dahliae*. Intron-exon boundaries and resulting open reading frames (ORF) were confirmed by PCR amplification and sequencing of wildtype cDNA. (**a**) Presumed architecture of the *V. dahliae* MAPK module including Ham5 scaffold. (**b**) Transcript structures and deduced protein kinase domains of *V. dahliae* MAPK2 Mek2 (top) and MAPK Vmk1 (bottom). The *MEK2* ORF results in 522 amino acids (aa) (blue: protein kinase domain 67–332 aa; IPR000719; grey: active serine/threonine site residues 186–198 aa, IPR008271; red: ATP binding site 73–96 aa; IPR017441). Vmk1 is smaller and consists of 355 aa (blue: protein kinase domain 23–311 aa, IPR000719; grey: active site serine/threonine residues 143–155 aa, IPR008271; red: ATP binding site 29–53 aa; IPR017441). (**c**) *HAM5* (*VDAG_JR2_Chr4g07170a*) transcripts and deduced protein domains. The 1553 aa Ham5 scaffold contains WD40 repeats at the N-terminus (black: 10–265 aa, IPR015943) and a coiled-coil domain (blue: 1154–1182 aa). Related Ham5-like proteins of other fungi are depicted in a phylogenetic tree (ClustalW algorithm) with *Trichoderma reesei* HAM-5 (AKN58846.1), *Neurospora crassa* HAM-5 (XP_011393509.1), *Podospora anserina* IDC1 (ABJ96338.2), *Aspergillus nidulans* HamE (AN2701), and *Saccharomyces cerevisiae* Ste5 (NP_010388.1) (Scale bar = average number of amino acid substitutions per site).

The unfolded protein response participates in cross regulation with the pheromone response MAPK pathway in the dimorphic plant pathogen *U. maydis* [58]. Pheromone response MAPK pathways integrate the sensing of extracellular signals and activate downstream pathways that control pathogenic differentiation and fungal virulence for numerous fungi including *V. dahliae* [51]. Pathway components are often assembled by scaffold proteins to maintain specificity and prevent crosstalk between signaling pathways. To date, scaffold proteins have not yet been analyzed in plant pathogenic fungi and it is unknown whether they are required for pheromone response MAPK pathway-mediated virulence. Therefore, we characterized the potential scaffold candidate Ham5 in *V. dahliae* (Figure 6a).

*V. dahliae HAM5* was identified by reciprocal BLASTp analysis and the deduced protein shows 53% amino acid sequence identity with the *N. crassa* scaffold homolog *HAM-5*. The 4906 bp *HAM5* pre-mRNA encodes a 1553 aa protein containing N-terminal WD40 repeats and a coiled-coil domain at the C-terminus. The corresponding proteins of related filamentous fungi are similar in length and show conserved protein domains. *V. dahliae MEK2* encodes a 522 aa MAP2K and *VMK1* encodes a 355 aa MAPK. Both proteins contain ATP binding and serine/threonine-protein kinase active sites (Figure 6b).

**Figure 7:**
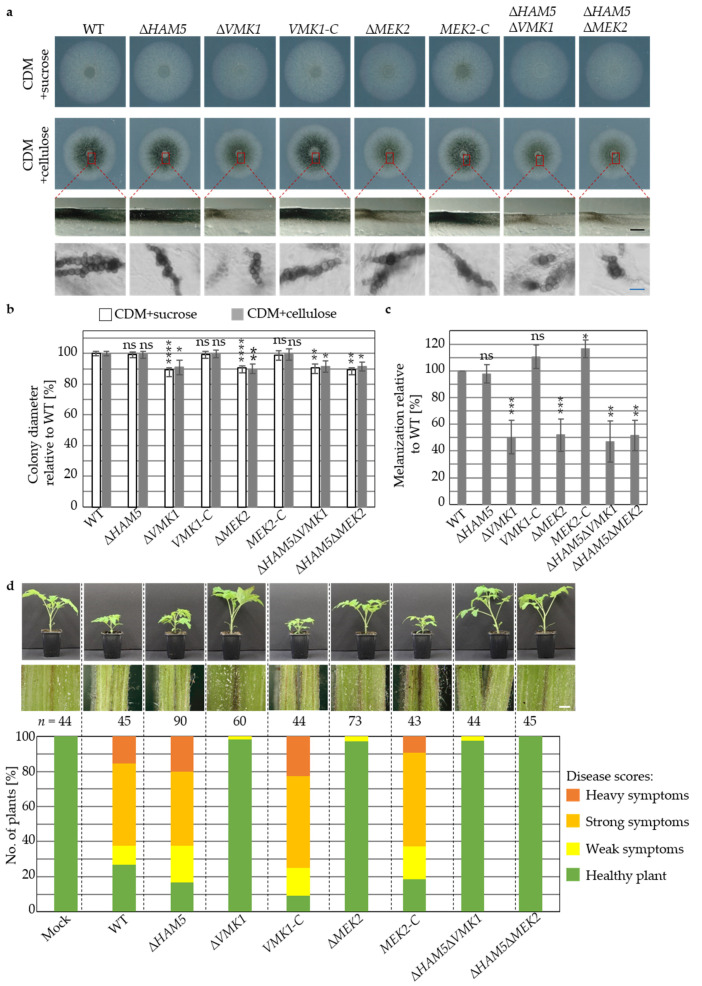
*Verticillium dahliae* Vmk1/Mek2 kinases, but not the Ham5 scaffold, are required for efficient melanization and virulence. The *HAM5* deletion strain (∆*HAM5*) was compared to wildtype (WT), *VMK1* and *MEK2* single deletions (∆*VMK1*; ∆*MEK2*) with respective complementation strains (*VMK1-C*; *MEK2-C*), as well as ∆*HAM5*∆*VMK1* and ∆*HAM5*∆*MEK2* double deletions. (**a**) Vegetative growth and melanization ex planta. Phenotypes were examined 9 d after spot inoculation of 50,000 freshly harvested spores on CDM with either sucrose or cellulose and incubation at 25 °C. Colony center cross-sections (red boxes/dashed lines) and microscopy of scraped fungal material (bottom) are shown for colonies grown on CDM with cellulose (black scale bar = 1 mm, blue scale bar = 20 µm). (**b**) Growth quantification 9 d after spot inoculation on CDM with sucrose or cellulose. ∆*HAM5* displays wildtype-like growth, whereas other single and double deletion strains are slightly repressed in growth. Mean values of three independent experiments relative to wildtype with standard deviations are shown (significant difference to wildtype on respective media: * *p* < 0.05; ** *p* < 0.01; **** *p* = 0, *n* ≥ 3). (**c**) Melanization of colonies 9 d after spot inoculation on CDM with cellulose. ∆*HAM5* displays wildtype-like melanization, whereas other single and double deletion strains display a decrease to about 40%. Mean values of two independent experiments relative to wildtype with standard deviations are shown (significant difference to wildtype: * *p* < 0.05; ** *p* < 0.01; *** *p* < 0.001; ns = non-significant, *n* ≥ 3). (**d**) Pathogenicity test of MAPK pathway mutant strains towards *Solanum lycopersicum*. Representative plants (top) and hypocotyl dissections (middle) 21 d after root dipping into distilled water as control (Mock) or the same numbers of spores obtained from different strains are shown (Scale bar = 1 mm). The stack diagram represents the relative number of plants with certain disease scores (total number of evaluated plants = *n*). Δ*HAM5* induces wildtype-like disease symptoms, whereas ∆*MEK2*, ∆*VMK1*, ∆*HAM5*∆*MEK2*, or ∆*HAM5*∆*VMK1* treated plants were comparable to mock plants and show no hypocotyl discolorations.

The role of the scaffold Ham5 in the Vmk1 MAPK pathway for fungal development and virulence was compared to Mek2 and Vmk1 through evaluation of the corresponding single and double gene deletion strains (Figure 7).

The *HAM5* deletion strain revealed wildtype-like growth and resting structure formation ex planta under stress and non-stress conditions, exemplified for CDM with sucrose or cellulose nine days after inoculation (Figure 7a). *MEK2* and *VMK1* single and double deletion strains with *HAM5* exhibited a 10% decrease in growth nine days after inoculation, whereas the complementation strains *MEK2-C* and *VMK1-C* displayed wildtype-like colony sizes (Figure 7a,b).

In addition, ∆*MEK2*, ∆*VMK1*, ∆*HAM5*∆*MEK2*, and ∆*HAM5*∆*VMK1* strains formed less microsclerotia on minimal medium with cellulose and melanization of the colonies was about 40% reduced, whereas ∆*HAM5* displayed wildtype-like melanization (Figure 7a,c). Ectopic integration of the corresponding wildtype gene in *MEK2-C* and *VMK1-C* strains resulted in wildtype-like phenotypes.

The impact of *V. dahliae* Ham5 was investigated in tomato plant infection experiments and compared to the core MAPK components Mek2 and Vmk1. Symptoms of tomato plants inoculated with spores from ∆*MEK2*, ∆*VMK1*, ∆*HAM5*∆*MEK2*, or ∆*HAM5*∆*VMK1* strains were comparable to mock plants after 21 days and no hypocotyl discolorations were observed (Figure 7d). The avirulent in planta phenotypes were complemented in experiments with *VMK1-C* and *MEK2-C*. Disease symptoms induced by the Δ*HAM5* strain were indistinguishable from the wildtype control and plants displayed severe stunting and discoloration of the vascular tissue.

These data corroborate that the requirement of the MAPK cascade components Vmk1 and Mek2 for virulence, growth and microsclerotia formation is independent of the presence of Ham5 and its predicted isolation function as scaffold protein in *V. dahliae*. These results highlight the potential for crosstalk between UPR and this MAPK signaling pathway, given that both promote fungal virulence, growth, and microsclerotia formation.

### 3.5. The Dual-Specificity Phosphatase Rok1 Limits V. dahliae Resting Structure Development and Promotes Growth and Conidiation

Regulation of MAPK activity by phosphatases appears to be a general feature in eukaryotes that is conserved in unicellular and filamentous fungi [93,94,95]. To date, the pheromone response MAPK dephosphorylating function of the yeast phosphatase Msg5 orthologues from the plant pathogenic fungi *M. oryzae* and *U. maydis* have been shown [96,97,98]. The orthologous phosphatase Rok1 in *U. maydis* is regulated and induced by the UPR, therefore connecting both signal pathways [58]. The corresponding *V. dahliae* orthologue was identified and consequences of the deletion were analyzed to decipher functions of the encoded phosphatase in this ascomycete.

*V. dahliae* Rok1 (*VDAG_JR2_Chr7g08960a*) with 803 aa harbors a dual-specificity phosphatase domain (337–528 aa, IPR020422) for removal of tyrosine or threonine phosphorylation [99] and shows high similarity to corresponding proteins of filamentous ascomycetes as *M. oryzae*, whereas it is more distantly related to *U. maydis* Rok1 or *S. cerevisiae* Msg5 (Figure S8).

**Figure 8:**
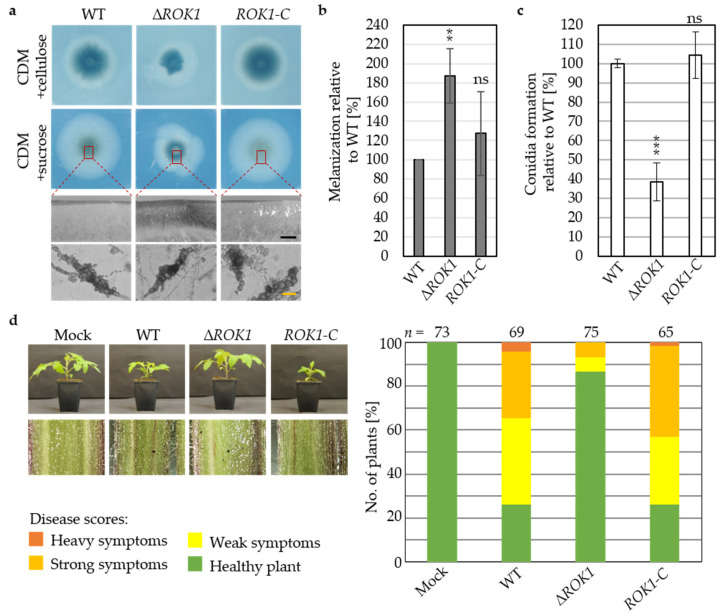
The *Verticillium dahliae* MAPK phosphatase Rok1 reduces microsclerotia formation and positively controls fungal growth and conidiation. The *ROK1* deletion strain (∆*ROK1*) was compared to wildtype (WT) and complementation strain (*ROK1-C*). (**a**) Vegetative growth and melanization ex planta. Phenotypes were examined 10 d after spot inoculation of 50,000 freshly harvested spores on CDM with either sucrose or cellulose and incubation at 25 °C. Colony center cross-sections (red boxes/dashed lines) and microscopy of scraped fungal material are shown for colonies grown on CDM with sucrose (black scale bar = 200 μm, yellow scale bar = 20 µm). (**b**) Melanization of colonies 10 d after spot inoculation on CDM with sucrose. ∆*ROK1* displays an increase in melanization about 80% relative to wildtype. Mean values of three independent experiments relative to wildtype with standard deviations are shown (significant difference to wildtype: ** *p* < 0.01; ns = non-significant, *n* = 3). (**c**) Quantification of conidiation in ∆*ROK1*. ∆*ROK1* conidiation 5 d post inoculation of liquid simulated xylem is reduced to about 60% relative to wildtype. Mean values of three independent experiments relative to wildtype with standard deviations are shown (significant differences to wildtype: *** *p* < 0.001, ns = non-significant, *n* = 3). (**d**) Pathogenicity test of *ROK1* deletion and complementation strains towards *Solanum lycopersicum*. Representative plants and hypocotyl dissections 21 d after root dipping into distilled water as control (Mock) or the same numbers of spores obtained from different strains are shown (Scale bar = 1 mm). The stack diagram represents the relative number of plants with certain disease scores (total number of evaluated plants = *n*). Δ*ROK1* induces less severe hypocotyl discolorations and overall disease symptoms in only 14% of treated plants.

A *V. dahliae ROK1* deletion strain was constructed, and the strain was used for in locus reintegration of the *ROK1* gene (*ROK1-C*). Fungal growth and development of the *ROK1* deletion strain were analyzed. ∆*ROK1* colonies appeared in asymmetric shape and qualitatively smaller on all tested media as exemplified by CDM with sucrose or cellulose ten days after spot inoculation (Figure 8a). Increased microsclerotia formation in the colony centers relative to wildtype was observed in cross sections (Figure 8a) and an increase in melanization of about 80% was quantified for colonies grown on minimal medium (Figure 8b). Compared to wildtype, ∆*ROK1* displayed about 60% reduction of conidia formed in liquid simulated xylem medium after five days (Figure 8c). The impact of *V. dahliae* Rok1 on plant pathogenicity was investigated. Symptoms of ∆*ROK1*-treated tomato plants displayed less severe stunting and hypocotyl discolorations in comparison to wildtype-infected plants. Disease symptoms were scored for only about 14% of ∆*ROK1*-treated plants (Figure 8d). The complementation strain *ROK1-C* displayed wildtype-like phenotypes.

Hence, the *V. dahliae* phosphatase Rok1 positively controls fungal growth, conidiation, and virulence, but restricts the formation of microsclerotia to an intermediate level. Accordingly, *ROK1* has the opposite impact on the formation of microsclerotia compared to the MAPK Vmk1 and the MAP2K Mek2, which exhibit positive effects on microsclerotia formation. This result corresponds to the idea that Rok1 controls the pheromone response MAPK pathway activity. To test this hypothesis, phosphorylation of the MAPK Vmk1 as putative target of the dual-specificity phosphatase Rok1 was tested using an α-phospho-p44/42 antibody that specifically detects the phosphorylated TEY motif of MAPKs in the *ROK1* deletion strain. The signal intensities for phosphorylated Vmk1 were comparable in protein extracts from wildtype, *ROK1* deletion, and complementation strain grown in minimal nutrient conditions. We examined the phosphorylation status of Vmk1 under UPR-induced conditions by supplementation of tunicamycin as an ER stress-inducing agent to test whether UPR induction regulates Rok1 activity and results in altered ratios of phosphorylated Vmk1. Activation of the UPR did not alter the previously observed picture of comparable signal intensities for phosphorylated Vmk1 (Figure S9). Therefore, we conclude that control of the activity of the dual-specificity phosphatase Rok1 towards dephosphorylation of the MAPK Vmk1 is independent of the tested minimal nutrient and UPR-induced conditions.

## 4. Discussion

**Figure 9:**
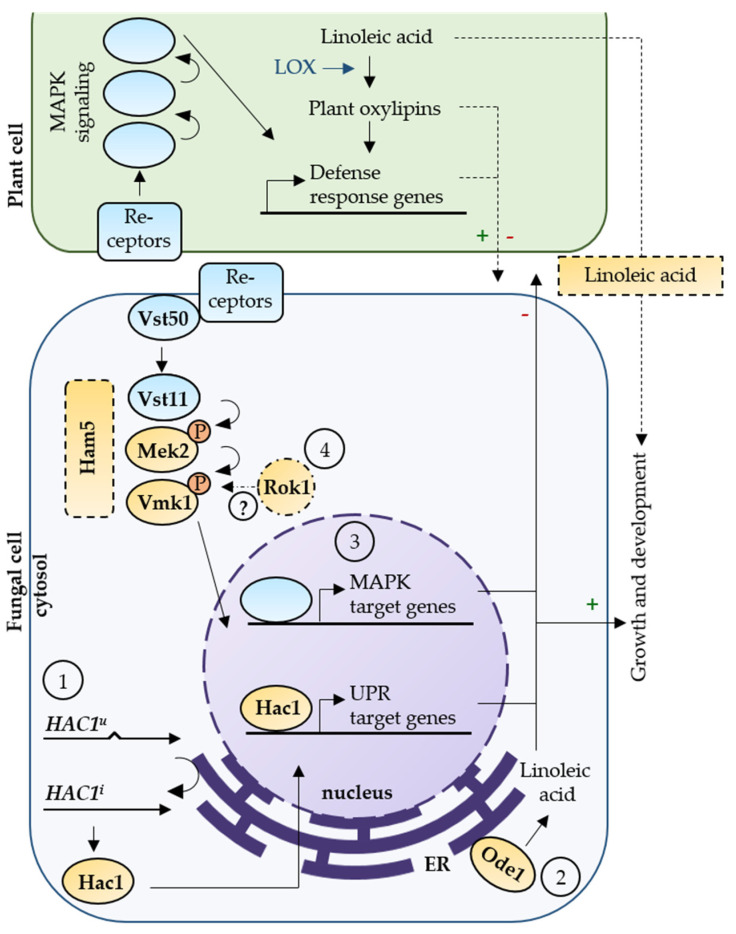
Model of *Verticillium dahliae* signaling pathways and of their functions in development and induction of disease symptoms. (1) The uninduced *HAC1* mRNA (*HAC1^u^*) is unconventionally spliced (arrow close to ER membrane), resulting in the basic leucine zipper transcription factor Hac1 encoding *HAC1^i^* mRNA. The translated Hac1 protein regulates UPR target genes. *V. dahliae* Hac1 has an impact on the response to ER stress, conidiation, vegetative growth even under non-stress conditions, and is essential for resting structure formation (arrow/green plus to “Growth and development”). Virulence of *V. dahliae* is strongly influenced by Hac1 (arrow/red minus from fungal cell), potentially by regulation of effector protein expression or secretion enabling circumvention of plant defense responses (arrow with dashed line/green plus from plant cell). (2) The *V. dahliae* oleate ∆12-fatty acid desaturase Ode1, catalyzing the linoleic acid biosynthesis, has a positive effect on vegetative growth (arrow/green plus to “Growth and development”) but only minor impact on disease symptom induction in the host. Linoleic acid provided by the plant might support fungal growth and differentiation (arrow with dashed line/green plus from plant cell). (3) *V. dahliae* MAPK signaling components Mek2 (MAP2K) and Vmk1 (MAPK) display positive impacts on vegetative growth and the formation of microsclerotia (arrow/green plus to “Growth and development”) and are essential for disease symptom induction in the host (arrow/red minus from fungal cell) independently from the *V. dahliae* Ham5 scaffold homolog. (4) The dual-specificity MAPK phosphatase Rok1 targets the counterpart of the MAPK Vmk1 in *U. maydis* [58], but the exact molecular function in *V. dahliae* is yet elusive (dashed arrow).

The unfolded protein response and the Ham5 scaffold-independent pheromone response MAPK pathway form important distinct signaling hubs for *V. dahliae* plant pathogenicity. The UPR regulator Hac1 is essential for induction of resting structure formation and conidiation, the first contact with the plant roots, and propagation within the plant. The linoleic acid synthesizing oleate ∆12-fatty acid desaturase Ode1 primarily affects fungal growth with only a negligible contribution to virulence of *V. dahliae*. The pheromone response MAPK pathway does not require the scaffold Ham5 to support maturation and melanization of microsclerotia as well as prompting disease symptoms as severe stunting and discoloration of the vascular tissue. The dual-specificity phosphatase Rok1 promotes growth, conidiation, and therefore dissemination and virulence. At the same time Rok1 limits the formation of resting structures for duration. The interplay between these signaling pathway components in *V. dahliae* growth, development, and induction of plant disease is summarized in Figure 9.

The *V. dahliae* UPR regulator Hac1 has conserved as well as species-specific impacts on fungal differentiation and is important for virulence. The *V. dahliae HAC1* mRNA contains an unconventional intron with sequence and structural similarity to Ire1 spliced introns in other organisms. Only the Hac1 protein resulting from translation of unconventionally spliced mRNA, but not from the uninduced variant, produced sufficient protein amounts for detection by immunoblots. Translation of *HAC1^u^* mRNA might be blocked or result in an unstable protein due to similar mechanisms as described for other ascomycetes. In *S. cerevisiae* base-pairing interaction between the unconventional intron and the 5′UTR leads to inhibition of ribosomal translation [100,101]. Additionally, accelerated degradation of Hac1^u^ proteins was described in yeast [102]. Due to shortened intron length in *HAC1^u^* mRNA of filamentous ascomycetes, a similar mechanism for translation inhibition as in yeast is not possible [103]. Rather, an impact of upstream open reading frames on translational repression of unspliced *HAC1* mRNA was assumed [104,105], but studies in *Aspergillus niger* revealed a translation attenuation mechanism by base-pairing of the 5′UTR of *HAC1* mRNA with an inverted repeat sequence [103]. Truncation of the 5′UTR was described to foster ER stress-dependent translation of *HAC1* mRNA in different Aspergilli, *T. reesei*, and *A. brassicicola* [31,104,105].

*V. dahliae* is viable in the presence of constitutively induced *HAC1* mRNA and does not require the presence of the uninduced variant. In contrast, regulatory roles of the unspliced mRNA homolog and the deduced protein were proposed for *U. maydis* [57]. Here, overexpression of the induced mRNA resulted in UPR hyper-activation and lethality unless unspliced mRNA was present in the cell [57].

In *S. cerevisiae*, *U. maydis*, and *Cryptococcus neoformans* the UPR is not required for vegetative growth and sporulation in the absence of ER stress [24,57,106,107]. In contrast, deletion of *HAC1* or genomic integration of the unconventionally spliced *HAC1* mRNA variant affects growth and conidiation without stress in different filamentous ascomycetes, including plant pathogens [30,31,103,108]. A basal UPR activity under non-stress conditions was determined in several fungi [15]. Constitutive activation of the UPR might alter the control of genes involved in growth and developmental processes [15]. The *V. dahliae HAC1* deletion strain was also generally impaired in growth and was not additionally affected in response to tunicamycin. The ability to cope with ER stress was improved upon expression of the induced *HAC1* splice variant, regardless of lower expression levels in comparison to wildtype. The *HAC1^i^-HA* strain displayed reduced growth under non-stress conditions. This might be caused by differential regulation of genes involved in growth in response to a hyper-active UPR. Supporting this idea, the induced mRNA variant of the UPR regulator was amplified from cultures grown under non-stress conditions in *V. dahliae*.

*HAC1* of the vascular pathogen *V. dahliae* is not only required for growth and conidiation in the absence of typical ER stress inducing conditions, but is also essential for the formation of microsclerotia. *HAC1* deletion strains do not form these resting structures under any tested condition. Decreased expression of the ectopically integrated *HAC1* gene lacking the conventional intron in the deletion background was sufficient to complement the microsclerotia phenotype in *HAC1^u^-HA*. For *HAC1^i^-HA* increased microsclerotia formation was observed. With *HAC1* being essential for the production of microsclerotia, the UPR might represent a checkpoint to induce resting structure formation in response to sensing unfavorable conditions. By which mechanisms this process is activated in *V. dahliae* is not yet understood, even if several candidates were shown to influence microsclerotia production such as the pheromone response MAPK pathway components Vmk1 and Mek2. An essential protein for microsclerotia formation is the transcription factor Som1 [109]. Som1 is involved in regulation of a subset of genes, such as the *Verticillium* transcription activators of adhesion Vta2 and Vta3, which have impacts on adhesion and microsclerotia formation [69,109]. Hac1 is involved in flocculin gene regulation and interacts with the general control of amino acid biosynthesis in *S. cerevisiae* [110]. Increased flocculation was observed for ER stressed yeast cells [111]. The findings that Som1 and Hac1-deficient *V. dahliae* strains don´t produce microsclerotia hint to an interconnected regulation between Som1 and the UPR during microsclerotia formation.

The *U. maydis* Hac1 orthologue is involved in induction of biotrophic growth in planta [33]. In contrast, UPR components are required for initial plant surface penetration in *M. oryzae* [30,51,112]. The *V. dahliae* UPR regulator Hac1 has a major impact on the fungal ability to colonize host plants and *HAC1* is required for efficient colonization of the root surface as first step. The subsequent penetration and initial root cortex invasion does not require Hac1, which is reminiscent of the finding that initial penetration of the plant surface was unaffected in the appressorium-forming fungi *A. brassicicola* and *U. maydis* [31,33]. Similar observations were made for the regulatory velvet protein Vel1 in *V. dahliae*, which is required for root colonization and disease symptom induction. Additionally, Vel1 is also involved in conidiospore and microsclerotia production as well as the regulation of secondary metabolism [113].

*V. dahliae HAC1* deletion strain treated tomato plants displayed severely decreased disease symptoms and fungal re-isolation from stems was not possible. Successful propagation within the host requires fungal spreading within the vascular system by conidiation, which is defective in the *HAC1* deletion strain. In addition, the UPR considerably contributes to the necessary adaptation of secretory capacities during host colonization of pathogens and the processing and secretion of fungal effectors in *U. maydis* [34,35,57,114]. The analysis of the *V. dahliae* secretome control by the Hac1 UPR regulator displays an interesting objective for future research.

Biosynthesis and secretion of lipid metabolites involved in growth, differentiation, and virulence of pathogenic fungi might be controlled by the ER homeostasis moderating UPR. In several cases, the virulence of plant pathogenic fungi depends on oxylipin signaling [48]. Characterization of the *V. dahliae* linoleic acid synthesizing oleate Δ12-fatty acid desaturase Ode1 showed an important impact on vegetative growth with only minor contribution to disease symptom induction. Likewise, decreased growth was ascertained after deletion of *A. nidulans* and *Aspergillus parasiticus* oleate Δ12-fatty acid desaturase homologs [44,45,46]. *V. dahliae ODE1* is dispensable for formation of wildtype-like resting structures, whereas a loss of sclerotia development was observed upon deletion of the *A. parasiticus ODE1* homolog [45,46].

Despite its impact on growth, *V. dahliae* induces severe disease symptoms in tomato plants independently of Ode1. The *V. dahliae ODE1* deletion strain displayed a minor growth defect on medium supplemented with linoleic acid or medium prepared from plant material supporting the idea that plant-derived unsaturated fatty acids can be used to compensate for the growth defect. Plant linoleic acid and the derived oxylipins are recognized as mimics of fungal signaling molecules and promote sporulation and mycotoxin production in Aspergilli [46,87,89,115,116,117,118].

Sensing of extracellular signals and activation of downstream differentiation and virulence regulating pathways is mediated by the pheromone response MAPK pathway in several unicellular and filamentous fungi including *V. dahliae* [51]. Different MAPK pathways can share components, like MAP kinases, adaptor proteins, or upstream kinases. For certain cascades scaffold proteins are necessary to bring components in proximity and maintain pathway specificity by isolation [119,120]. Scaffold proteins are described for the pheromone response MAPK pathway in different filamentous ascomycetes and support fusion of fungal cells [59,60,61]. The *N. crassa* scaffold protein HAM-5 assembles the MAPK cascade during chemotropic growth and positively influences growth and differentiation [59,60,121,122]. The homologous *A. nidulans* scaffold protein HamE is required for sexual and asexual development and secondary metabolite production [61]. The role of homologous scaffold proteins was yet unstudied in plant pathogens. Our findings demonstrate that pheromone response MAPK cascade-mediated growth, differentiation, and virulence are independent from isolation of this pathway by the Ham5 scaffold protein in *V. dahliae*.

In addition to scaffold proteins, dual-specificity MAPK phosphatases control MAPK signal forwarding by dephosphorylation of the final MAPK and therefore reduction of pathway activity. The putative pheromone response MAPK phosphatase Rok1 in *V. dahliae* has positive impacts on fungal growth and conidiation and contrary limits the formation of microsclerotia to an intermediate level. In contrast, the *V. dahliae* MAPK Vmk1 and the MAP2K Mek2 display positive impacts on microsclerotia formation. The opposite functions of the pheromone response MAPK pathway components and the putative phosphatase support the hypothesis that Rok1 controls the pheromone response MAPK pathway activity. A cross-regulation of these pathways via the dual-specificity MAPK phosphatase Rok1 has been shown in the basidiomycete *U. maydis* [58]. Deletion of *ROK1* did not alter the phosphorylation status of the MAPK Vmk1 under tested conditions, which suggests that Rok1 might act on different yet unidentified target proteins.

*M. oryzae*, *Fusarium graminearum*, and *F. oxysporum* mutant strains defective in the orthologous phosphatase gene displayed reduced growth as well as reduced virulence on their host plants [123,124,125,126]. An additional effect on conidiation was observed for *M. oryzae* and *F. oxysporum* [123,125]. In these fungi, the phosphatase was shown to act on the cell wall integrity MAPK pathway. An additional dephosphorylation activity on the pheromone response MAPK was only shown for the *M. oryzae* protein. The observed phenotypes resemble our phenotypical observations in *V. dahliae*. Whether *V. dahliae* Rok1 controls activity of the cell wall integrity MAPK pathway remains to be elucidated. Components of this MAPK pathway have not yet been characterized in *V. dahliae* and functions on fungal differentiation are still unknown. However, upregulation of the cell wall integrity MAP2K and MAPK encoding orthologues in later stages of microsclerotia formation was shown, similar to Vmk1 and Mek2 encoding genes [127].

Our results show that different signaling cascades, including the secretion capacity moderating UPR, the biosynthesis of linoleic acid as precursors of oxylipins, the scaffold-independent pheromone response MAPK pathway, and the dual-specificity phosphatase Rok1 contribute to fungal growth. Further, the MAPK pathway, the phosphatase Rok1, and UPR are required for induction of Verticillium wilt in the host plant. Additionally, both pathways as well as the phosphatase Rok1 are involved in regulation of microsclerotia development and, therefore, for persistence of the fungus under unfavorable environmental conditions. The similarities of the signaling component mutant phenotypes in *V. dahliae* support an interplay between the secretion capacity moderating UPR and the scaffold-independent pheromone response MAPK pathway in regulation of fungal differentiation and virulence. This interplay results in promotion or reduction of fungal growth and influences the outcome of the interaction of *V. dahliae* with its host plant. The Rok1 phosphatase control function in the ascomycete *V. dahliae* might address additional targets than the direct interplay between UPR and the MAPK, which is described for the basidiomycete *U. maydis* [58]. Further analyses are required to substantiate the molecular level of the connection between these pathways, which represent promising targets to control growth, survival of microsclerotia in the soil, and propagation in the plant as a means to reduce the devastating impact on crops of this important vascular pathogen.

## Data Availability

Data and Images supporting reported results presented in Figures and Supplementary Figures of the manuscript are available as Supplementary Materials as Data S1 and Images S1.

## Supplementary Materials

The following are available online at https://www.mdpi.com/article/10.3390/jof7040305/s1, Methods S1; Figure S1: Southern hybridization of V. dahliae HAC1 deletion, complementation, as well as HAC1u-HA and HAC1i-HA strains; Figure S2: Southern hybridization of V. dahliae ODE1 deletion, ODE1-GFP complementation, and ODE1 deletion strain with ectopically integrated GFP; Figure S3: Southern hybridization of V. dahliae HAM5, VMK1, and MEK2 single and double deletion and complementation strains; Figure S4: Southern hybridization of V. dahliae ROK1 deletion and ROK1-C complementation strains. Figure S5: The cDNA sequence of V. dahliae JR2 HAC1i. Figure S6: The amino acid sequence of V. dahliae JR2 Hac1. Figure S7: V. dahliae ∆ODE1 has only a minor effect on vegetative growth on plant agar. Figure S8: Phylogeny of Rok1-like phosphatases; Figure S9: Vmk1 phosphorylation is unaffected upon deletion of ROK1 in V. dahliae. Table S1: Verticillium strains constructed and used in this study; Table S2: Primers used in this study; Table S3: Plasmids constructed and used in this study; Data S1; Images S1.
