# Supplementary material for: Unfolded Protein Response and Scaffold Independent Pheromone MAP Kinase Signaling Control Verticillium dahliae Growth, Development, and Plant Pathogenesis
Source: J Fungi (Basel). 2021 Apr 15;7(4):305. doi: 10.3390/jof7040305 (PMC8071499; doi:10.3390/jof7040305)
Supplement: Supplementary file 1 [file jof-07-00305-s001.zip › Images_S1.pdf]

Supplementary Material

Supplementary Images S1

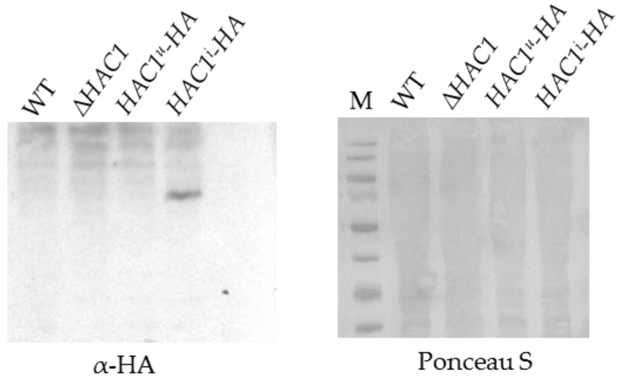

Images Figure 2: Immunoblot of *V. dahliae* Hac1 proteins in *HAC1*^{u}*-HA* and *HAC1*^{i}*-HA*. M: PageRuler Prestained Protein Ladder 10–180 kDa (Thermo Fisher Scientific, Waltham, MA, USA).

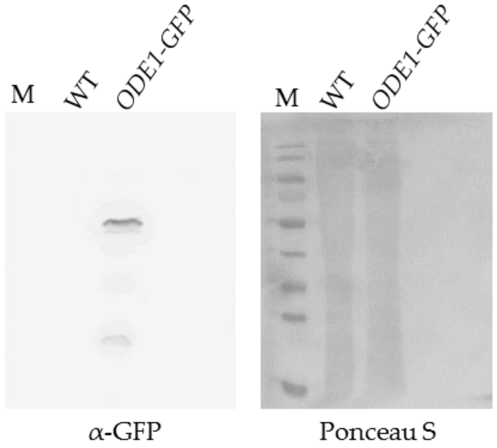

Images Figure 4: Immunoblot of *V. dahliae* Ode1-GFP. M: PageRuler Prestained Protein Ladder 10–180 kDa (Thermo Fisher Scientific, Waltham, MA, USA).

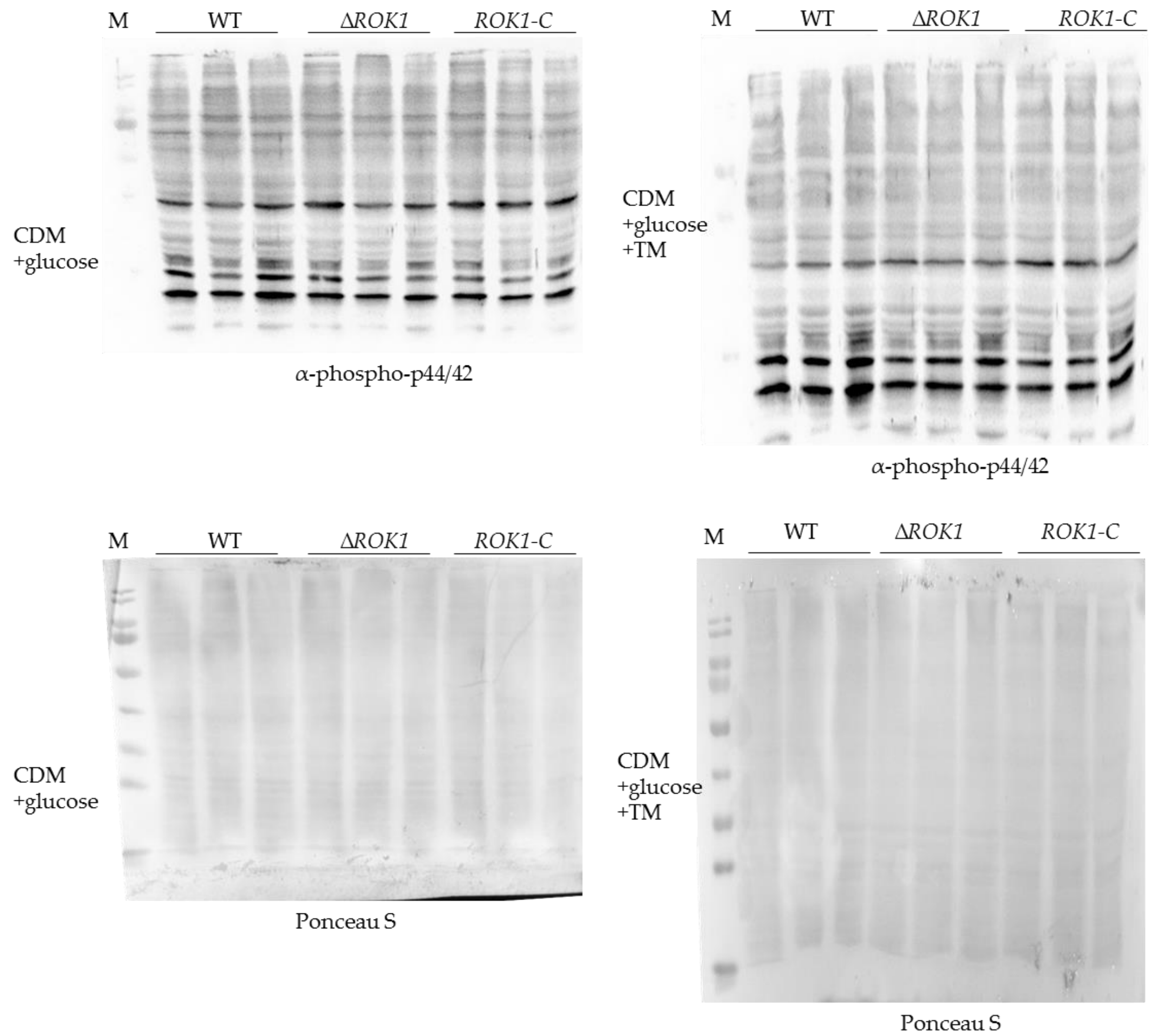

Images Figure S9: Immunoblots of *V. dahliae ROK1* deletion and *ROK1-C* complementation strains. M: PageRuler Prestained Protein Ladder 10–180 kDa (Thermo Fisher Scientific, Waltham, MA, USA).
