# Supplementary material for: Unfolded Protein Response and Scaffold Independent Pheromone MAP Kinase Signaling Control Verticillium dahliae Growth, Development, and Plant Pathogenesis
Source: J Fungi (Basel). 2021 Apr 15;7(4):305. doi: 10.3390/jof7040305 (PMC8071499; doi:10.3390/jof7040305)
Supplement: Supplementary file 1 [file jof-07-00305-s001.zip › Supplementary_Material.docx]

### Supplementary Material

### *Supplementary Methods*

**Methods S1**

**DNA manipulation and strain construction**

The gene predictions for *V. dahliae* JR2 *BIP1* (*VDAG_JR2_Chr3g10940a*), *ODE1* (*VDAG_JR2_Chr1g29610a*), *HAM5* (*VDAG_JR2_Chr4g07170a*), *MEK2* (*VDAG_JR2_Chr1g13070a*), *VMK1* (*VDAG_JR2_Chr2g01260a*), and *ROK1* (*VDAG_JR2_Chr7g08960a*) were used from Ensembl Fungi (https://fungi.ensembl.org, [78]) and were confirmed on cDNA level. We verified the predicted *HAC1* (*VDAG_JR2_Chr2g09780a*) transcript variant as the uninduced variant *HAC1^u^* and identified a second splice variant of the *HAC1* mRNA named *HAC1^i^* (sequence given in Figure S5). To induce the formation of the transcript variants, different conditions were applied. Total RNA was isolated from wildtype cultures grown in 50 mL SXM (1 × 10^7^ spores) and incubated at 25 °C under constant agitation for 4 d for the uninduced *HAC1* mRNA variant and with subsequent supplementation with 3 mM DTT for 3 h for the induced *HAC1* mRNA variant. The cDNA was generated and used for amplification of *HAC1*. The reverse transcribed 1581 bp uninduced *HAC1* mRNA sequence and the 1254 bp induced splice variant were amplified with primers JST171/JST172 and JST171/JST174, respectively. Transcripts were fully sequenced. For PCR amplifications the Q5 Hot Start polymerase (New England Biolabs, Ipswich, MA, USA) or Phusion polymerase (Thermo Fisher Scientific, Waltham, MA, USA) were used. Fungal transformants were verified by Southern hybridization [109].

The GeneArt Seamless Cloning and Assembly Kit (Thermo Fisher Scientific, Waltham, MA, USA) and the FastCloning protocol [74] were used to construct deletion and complementation cassettes. From *V. dahliae* JR2 genomic DNA up- and downstream flanking sequences were amplified with 15‑16 bp homologous overhangs to the desired neighboring fragments. NucleoSpin Gel and PCR Clean-up Kits (Macherey-Nagel, Düren, Germany) were used for purification of PCR products. *E. coli* DH5α cells (Invitrogen, Carlsbad, CA, USA) were transformed with the cloned constructs. All plasmids were verified by Sanger sequencing performed by the Microsynth Seqlab in Göttingen.

Plasmid and strain construction of the *HAC1* deletion

For construction of the *HAC1* deletion cassette, primer pair JST186/JST187 was used to amplify a 1500 bp flanking region 218 bp upstream from the ORF and a 1445 bp 3′-flanking region was amplified with JST188/JST189 from fungal wildtype DNA. The 3942 bp *HYG^R^* marker cassette was amplified from pPK2 [70] with primers ML8/RO3. The fragments were ligated to pME4564 [71] linearized with *Eco*RV and *Stu*I. Resulting pME4830 was used for wildtype transformation. For Southern hybridization, genomic DNA was cut with *Sal*I and amplified 300 bp *HAC1* 3′-flanking region (JST272/JST273) was labelled as a probe. The verified *HAC1* deletion transformants were conserved as VGB371 and VGB372.

Plasmid and strain construction of the ectopic *HAC1* complementation

For construction of the ectopic *HAC1* complementation cassette, primers JST216/JST211 were used to amplify a 3636 bp insert containing 1702 bp 5′-flanking, 1634 bp *HAC1* gene, and 300 bp 3′-flanking region. This fragment was ligated to *Eco*RV-linearized pME4815 [71]. The resulting plasmid pME4831 was used for ∆*HAC1* transformation. For Southern hybridization the same probe and restriction enzyme as for the *HAC1* deletion strain were used. The verified *HAC1-C* transformant was conserved as VGB382.

Plasmid and strain construction of the ectopic *HAC1^u^*-*HA* complementation

For construction of a *HAC1^u^-HA* complementation construct, primers JST171/JST172 were used to amplify the uninduced mRNA splice variant of *HAC1* without stop codon from cDNA isolated from wildtype cultures incubated in SXM for 4 d. The C-terminal *HA*-tag was fused to *HAC1^u^* via amplification with JST171/JST267 including a 48 bp *HA* sequence as overhang. *HAC1^u^-HA* was ligated to the cloning vector pJet1.2, resulting in the plasmid pME4832.

The 1400 bp 5′-flanking and the 300 bp 3′-flanking region were amplified from fungal wildtype DNA with primers JST269/JST270 and JST272/JST273, respectively. The 1626 bp *HAC1^u^-HA* sequence was amplified from pME4832 with JST171/JST268. The fragments were ligated to *Eco*RV-linearized pME4815 [71]. The resulting plasmid pME4834 was used for ∆*HAC1* transformation. For Southern hybridization the same probe and restriction enzyme as for the *HAC1* deletion strain were used. The verified *HAC1^u^-HA* transformants were conserved as VGB439 and VGB440.

Plasmid and strain construction of the ectopic *HAC1^i^*-*HA* complementation

For construction of a *HAC1^i^-HA* complementation construct, the primer pair JST171/JST174 was used to amplify the induced splice variant of *HAC1* without stop codon from cDNA, isolated from wildtype cultures incubated in SXM for 4 d with subsequent supplementation with 3 mM DTT for 3 h. The C‑terminal *HA*-tag was fused to *HAC1^i^* via amplification with JST171/JST266 including a 48 bp *HA* sequence as overhang. *HAC1^i^-HA* was ligated to the cloning vector pJet1.2, resulting in pME4833. The 1400 bp 5′-flanking region and the 607 bp 3′-flanking region were amplified from fungal wildtype DNA with primers JST269/JST270 and JST271/JST272, respectively. The 1299 bp *HAC1^i^-HA* sequence was amplified from pME4833 with JST171/JST268. The fragments were ligated to *Eco*RV-linearized pME4815 [71], resulting in pME4835 used for transformation of the ∆*HAC1* strain. For Southern hybridization, the same probe and restriction enzyme as for Δ*HAC1* was used. The confirmed *HAC1^i^-HA* transformants were conserved as VGB437 and VGB438.

Plasmid and strain construction of the *ODE1* deletion strain

For construction of the *ODE1* cassette, the 1000 bp 5′- and 1522 bp 3′-flanking regions were amplified from fungal wildtype DNA with primers JST127/JST128 and JST129/JST130, respectively. The 2194 bp *NAT^R^* marker cassette was amplified with ML8/ML9 from pME4815 [71]. All fragments were ligated to the 6728 bp pME4564 [71] backbone amplified with JST137/JST138. The resulting plasmid pME4836 was used for wildtype transformation. For Southern hybridization, genomic DNA was cut with *Sca*I and the amplified *ODE1* 3′-flanking region was labelled as a probe. The verified ∆*ODE1* transformants were conserved as VGB331 and VGB332.

Plasmid and strain construction of endogenous C-terminally *GFP*-tagged *ODE1* complementation

For construction of the endogenous *ODE1* complementation cassettes with C‑terminal *GFP*-tag, primers JST129/JST130 were used to amplify the 1522 bp 3′-flanking region from fungal wildtype DNA. This was ligated to the 10744 bp pPK2 [70] backbone amplified with JST138/JST177, resulting in plasmid pME4837. JST179/JST180 primer pair was used to amplify a 2501 bp PCR product containing the 1000 bp 5′-flanking region and 1501 bp *ODE1* gene without stop codon from fungal wildtype DNA. From pGreen2 [69] the sequence of 720 bp C-terminal *GFP* with a 15 bp linker was amplified with primers SAB16/JST178. Both inserts were ligated to *Eco*RV-linearized pME4837, resulting in plasmid pME4838 used for transformation of the ∆*ODE1* strain. For Southern hybridization, the same probe and restriction enzyme as for Δ*ODE1* was used. Additionally, the expression of the fusion protein was confirmed by fluorescence microscopy and immunoblotting with a GFP antibody. The verified transformants were conserved as VGB358 and VGB359 (*ODE1-GFP*).

Plasmid and strain construction of the ectopic *GFP* overexpression strains WT *OE-GFP^NAT^, ΔHAC1 OE-GFP*, and *ΔODE1 OE-GFP*

For construction of the *GFP* overexpression vector with *NAT^R^* marker cassette, primers JST184/JST185 were used to amplify a 2378 bp fragment containing *GFP* under control of the *gpdA* promoter and *trpC* terminator from pGreen2 [69]. This fragment was ligated to pME4815 [71], amplified with primers ML1/ML9, resulting in pME4819 used for wildtype and ∆*HAC1* transformation. The ∆*ODE1* strain was transformed with the plasmid pGreen2 [69].

The resulting WT *OE-GFP^NAT^* transformant was confirmed by fluorescence microscopy and phenotypic comparison to wildtype and conserved as VGB392.

The resulting ∆*HAC1* *OE-GFP* transformant was confirmed by fluorescence microscopy, phenotypic comparison to the ∆*HAC1* strain and Southern hybridization. For Southern hybridization, the same probe and restriction enzyme as for Δ*HAC1* was used. The verified ∆*HAC1* *OE-GFP* transformant was conserved as VGB380.

The resulting ∆*ODE1* *OE-GFP* transformant was confirmed by fluorescence microscopy, phenotypic comparison to the ∆*ODE1* strain and Southern hybridization. For Southern hybridization, the same probe and restriction enzyme as for Δ*ODE1* was used. The verified ∆*ODE1* *OE-GFP* transformant was conserved as VGB357.

Plasmid and strain construction of WT *Histone-RFP* and *ODE1-GFP Histone-RFP* strains

For visualization of nuclei, a plasmid with *RFP* fused to *H2B* (*VDAG_JR2_Chr2g01720a*) under the control of the *gdpA* promoter was generated. The *gpdA* promoter was amplified from pKO2.0 [71] using the primers PC4/RH523. The *RFP* fragment was generated from pME3857 [72] with the primers RH524/RH525. Genomic DNA from the wildtype JR2 served as template for *H2B* and the primers RH526/RH527 were used. The *trpC* terminator was amplified from pPK2 [70] using the primers RH528/RH529. The single fragments were fused by fusion PCR using the primers PC4/RH529. The fusion product was ligated into the pJet1.2 vector, resulting in pME4973. The *RFP-H2B* expression cassette was amplified from pME4973 using the primers pJet1.2 reverse/RH530. The fragment as well as the vector pPK2 [70] was restricted with *Xba*I and ligated, resulting in pME4975. To generate a plasmid with *GEN^R^* marker cassette, the *RFP-H2B* expression cassette was amplified from pME4975 using the primers JT1/JT2. The plasmid pCOM [73] was linearized with *Sma*I. The *RFP-H2B* expression cassette was inserted into the vector using the Seamless cloning and assembly kit, resulting in pME4976. This plasmid was used for transformation of the wildtype and VGB358, resulting in VGB477 and VGB493/VGB494.

Plasmid and strain construction of the *HAM5* single and double deletion with *VMK1*

For construction of the *HAM5* deletion cassette, the 1500 bp flanking region 333 bp upstream of the ORF and the 1000 bp downstream flanking region were amplified with primers JST110/JST111 and JST112/JST113, respectively, from wildtype fungal DNA. ML8/ML9 were used for amplification of the 2194 bp *NAT^R^* marker cassette from pME4815 [71]. Primers ML1/ML2 were used to amplify the 6728 bp backbone of pME4564 [71], which was ligated to the fragments resulting in the plasmid pME4820. This plasmid was used for wildtype and ∆*VMK1* (VGB335) transformation. For Southern hybridization, genomic DNA was cut with *Sac*I and the 3′-flanking region of *HAM5* was labelled as a probe. The *HAM5* single deletion transformants were conserved as VGB279 and VGB280. The *HAM5* and *VMK1* double deletion transformant was conserved as VGB417.

Plasmid and strain construction of the ectopic *HAM5* complementation

For construction of the ectopic *HAM5* complementation cassette, primers JST245/JST246 were used to amplify a 7265 bp sequence including 1359 bp 5′-flanking region, 4906 bp *HAM5* ORF, and 1000 bp 3′-flanking region from fungal genomic DNA. The PCR product was ligated into *Eco*RV-linearized pPK2 [70], resulting in the plasmid pME4828, which was used for ∆*HAM5* transformation. For Southern hybridization, the same probe and restriction enzyme as for Δ*HAM5* was used. The resulting *HAM5-C* transformant was conserved as VGB415.

Plasmid and strain construction of the *VMK1* deletion

For construction of the *VMK1* deletion cassette, primers JS-V21/JS-V22 were used to amplify the 898 bp 3′-flanking region from fungal wildtype DNA. This was ligated to the 10739 bp pPK2 [70] backbone generated with JS-V23/JS-V24, resulting in pME4824. From this plasmid the backbone was amplified with primers ML1/JS-V23 and ligated to the 1472 bp 5′-flanking region, which was generated with primers JST77a/JST76b from fungal wildtype DNA. The resulting plasmid pME4825 was used for wildtype transformation. For Southern hybridization, genomic DNA was cut with *Bgl*I and the 3′-flanking region of *VMK1* was prepared as a probe. The verified *VMK1* deletion transformants were conserved as VGB335 and VGB336.

Plasmid and strain construction of the ectopic *VMK1* complementation

For construction of the ectopic *VMK1* complementation cassette, primers JST243/JST244 were used to amplify a 3661 bp sequence, including 1473 bp 5′-flanking region, 1260 bp *VMK1* ORF, and 928 bp 3′-flanking region, from fungal wildtype DNA. The PCR product was ligated to *Eco*RV-linearized pME4815 [71], resulting in the plasmid pME4827 used for ∆*VMK1* transformation. For Southern hybridization, the same probe and restriction enzyme as for Δ*VMK1* was used. The resulting *VMK1-C* transformant was conserved as VGB413.

Plasmid and strain construction of the *MEK2* single and double deletion with *HAM5*

For construction of the *MEK2* deletion cassette, primers JS-V5/JS-V6 were used to generate the 1500 bp 5′-flanking region from fungal wildtype DNA. The fragment was ligated to the 10676 bp pPK2 [70] backbone amplified with JS-V7/JS-V8, resulting in pME4821. The backbone of this plasmid was generated with JS-V11/JS-V12 and ligated to the 1500 bp 3′-flanking region 385 bp downstream of the ORF, which was amplified with primers JS-V9/JS-V10 from fungal wildtype DNA. The resulting plasmid pME4822 was used for wildtype and ∆*HAM5* (VGB279) transformation. The 3′-flanking region of *MEK2* was amplified and labelled as a probe for Southern hybridization and genomic DNA was cut with *Nru*I. The verified *MEK2* single deletion transformants were conserved as VGB337 and VGB338. The *MEK2* and *HAM5* double deletion transformant was conserved as VGB346.

Plasmid and strain construction of the ectopic *MEK2* complementation

For construction of the ectopic *MEK2* complementation cassette, primers JST212/JST213 were used to amplify a 5176 bp sequence including 1500 bp 5′-flanking region, 1756 bp *MEK2* ORF, and 1885 bp 3′-flanking region from fungal wildtype DNA. The PCR product was ligated to pME4815 [71] cut with the restriction enzyme *Eco*RV, resulting in the plasmid pME4826 used for ∆*MEK2* transformation. The 3′-flanking region of *MEK2* was amplified and labelled as a probe for Southern hybridization and genomic DNA was cut with *Hind*III. The resulting *MEK2-C* transformants were conserved as VGB388 and VGB389.

Plasmid and strain construction of the *ROK1* deletion

For construction of the *ROK1* deletion cassette, a 1500 bp flanking region upstream from the ORF was amplified using JST314/JST315, and a 750 bp 3′-flanking region was amplified using JST316/JST317 from fungal wildtype DNA. The 1648 bp *GEN^R^* marker cassette was amplified from pCOM [73] with primers JST253/JST254. The fragments were ligated to pME4564 [71] cut with restriction enzymes *Eco*RV and *Stu*I, resulting in pME5092 used for wildtype transformation. The 3-flanking region of *ROK1* was labelled as a probe for Southern hybridization and genomic DNA was cut with *Hind*III. The resulting *ROK1* deletion transformant was conserved as VGB532.

Plasmid and strain construction of endogenous *ROK1* complementation

For construction of the endogenous *ROK1* complementation cassette with *NAT^R^*, the 1500 bp 5′‑flanking region was amplified together with the 3586 bp *ROK1* ORF from fungal wildtype DNA with primers JST314/JST319. The 750 bp 3′-flanking region was amplified with JST316/JST317 from fungal wildtype DNA. The 1955 bp *NAT^R^* was amplified with ML5/JST254 from pME4815 [71]. The inserts were ligated to pME4564 [71] cut with restriction enzymes *Eco*RV and *Stu*I, resulting in plasmid pME5093. For Southern hybridization the same restriction enzyme and probe were used as for Δ*ROK1*. The resulting *ROK1* complementation transformant was conserved as VGB548.

### *Supplementary Figures*

| **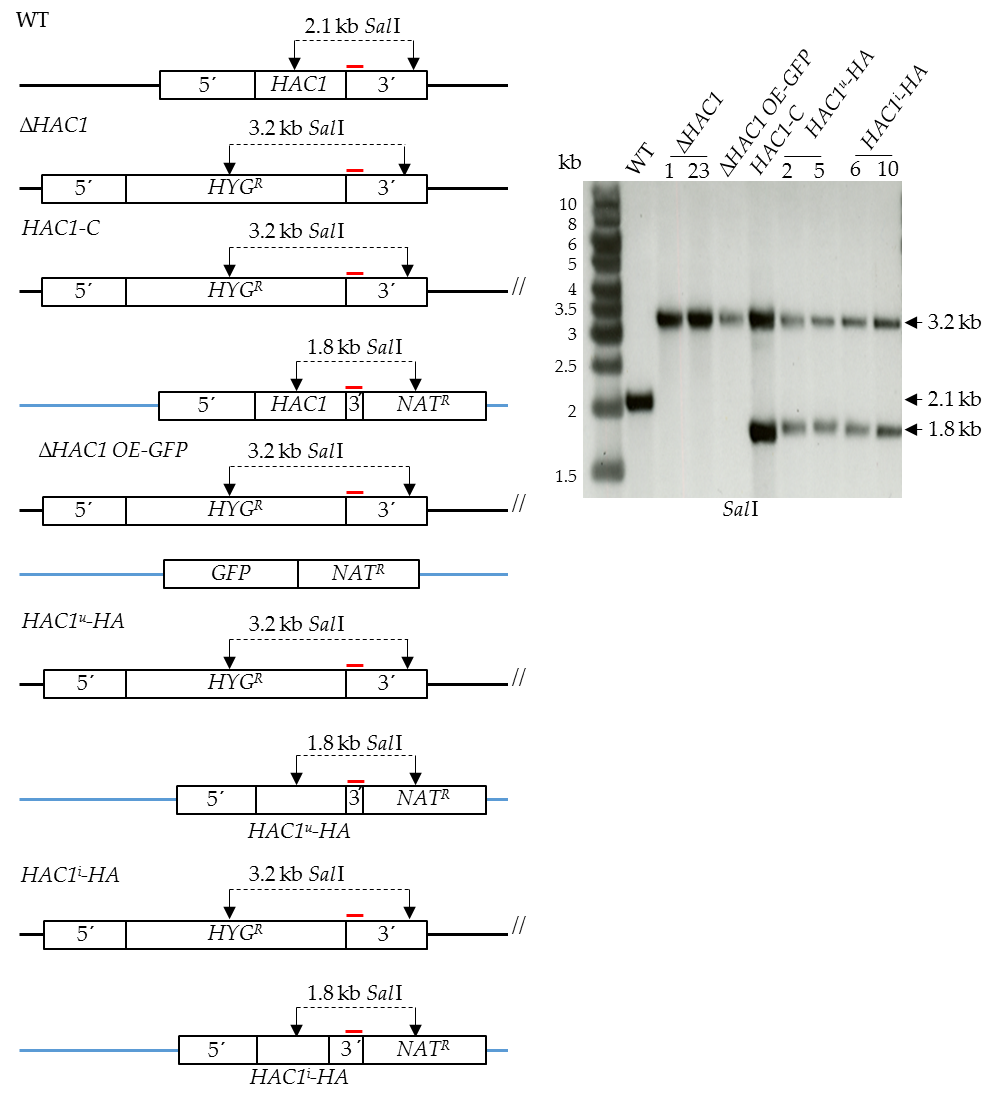** |
| --- |

**Figure S1. Southern hybridization of *V. dahliae* *HAC1* deletion, complementation, as well as *HAC1^u^‑HA* and *HAC1^i^‑HA* strains.** *V. dahliae* wildtype (WT), *HAC1* deletion (∆*HAC1* transformant number 1 and 23), complementation (*HAC1-C*) strains, and strains harboring either one of the two ectopically integrated *HAC1* mRNA splice variants (*HAC1^u^‑HA* transformant number 2 and 5; *HAC1^i^‑HA* transformant number 6 and 10), or the ectopically integrated *GFP* overexpression construct (∆*HAC1 OE-GFP*) were tested. *Sal*I was used to cut genomic DNA and 300 bp of the *HAC1* 3′ flanking region served as a probe. Left: Scheme of restriction sites (arrows) and expected fragment length. *HYG^R^*: hygromycin resistance cassette with *gpdA* promoter and a *trpC* terminator; *NAT^R^*: nourseothricin resistance cassette with *gpdA* promoter and a *trpC* terminator; //: end of endogenous locus; blue line: ectopic integration sites; red line: probe used for Southern hybridization. Right: Signals corresponding to 2.1 kb for wildtype, 3.2 kb for ∆*HAC1* and ∆*HAC1 OE‑GFP,* and 3.2 kb as well as 1.8 kb for the *HAC1-C,* *HAC1^u^-HA* and *HAC1^i^‑HA* strains were observed as predicted.

| **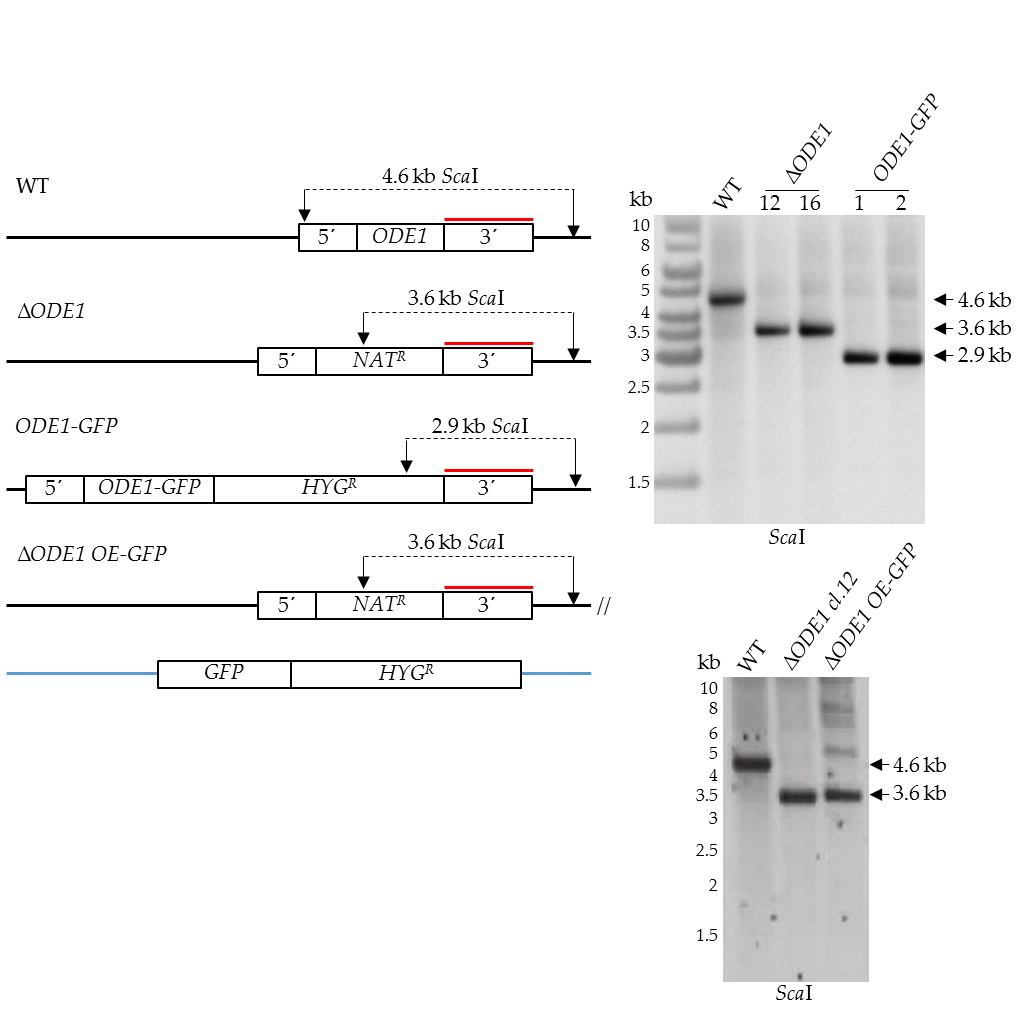** |
| --- |

**Figure S2. Southern hybridization of *V. dahliae ODE1* deletion****, *ODE1-GFP* complementation, and *ODE1* deletion strain with ectopically integrated *GFP*.** *V. dahliae* wildtype (WT), *ODE1* deletion (∆*ODE1* transformant number 12 and 16), the complementation strain harboring an *ODE1-GFP* construct under control of the native promoter and terminator at the endogenous locus (*ODE1-GFP* transformant number 1 and 2), and the *ODE1* deletion with ectopically integrated *GFP* were tested. *Sca*I was used to cut genomic DNA and the *ODE1* 3′ flanking region served as a probe (red line). Left: Scheme of restriction sites (arrows) and expected fragment length. *HYG^R^*: hygromycin resistance cassette with *gpdA* promoter and a *trpC* terminator; *NAT^R^*: nourseothricin resistance cassette with *gpdA* promoter and a *trpC* terminator; //: end of endogenous locus; blue line: ectopic integration sites. Right: Signals corresponding to 4.6 kb for wildtype, 3.6 kb for the deletion and 2.9 kb for the endogenous complementation strains were obtained as predicted.

| **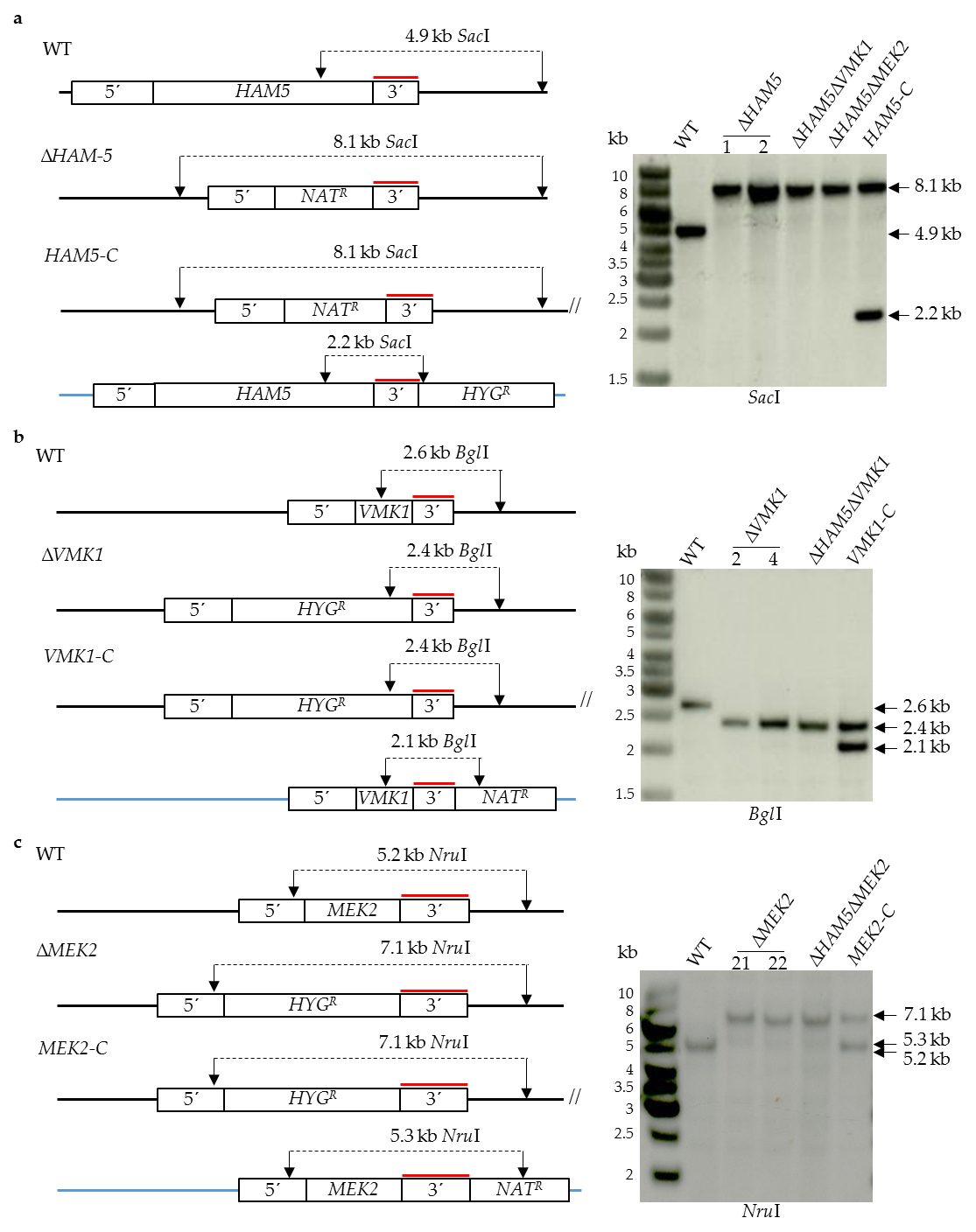** |
| --- |

**Figure S3. Southern hybridization of *V. dahliae* *HAM5, VMK1,* and *MEK2* single and double deletion and complementation strains.** *V. dahliae* wildtype (WT), *HAM5, VMK1,* and *MEK2* single (∆*HAM5* transformant number 1 and 2; ∆*VMK1* transformant number 2 and 4*;* ∆*MEK2* transformant number 21 and 22), double deletions (∆*HAM5*∆*VMK1;* ∆*HAM5*∆*MEK2*), and complementation (*HAM5-C; VMK1-C; MEK2-C*) strains were tested. *HYG^R^:* hygromycin resistance cassette with *gpdA* promoter and a *trpC* terminator; *NAT^R^*: nourseothricin resistance cassette with *gpdA* promoter and a *trpC* terminator; //: end of endogenous locus; blue line: ectopic integration sites; red line:  probes used for Southern hybridization. (**a**) Southern hybridization of *HAM5* single and double deletion and complementation strains with *Sac*I. Left: Scheme of restriction sites (arrows) and expected fragment length. Right: Signals corresponding to 4.9 kb for wildtype, 8.1 kb for the deletion, and 2.2 kb as well as 8.1 kb for the complementation were obtained as predicted. (**b**) Southern hybridization of *VMK1* single and double deletion and complementation strains with *Bgl*I. Left: Scheme of restriction sites (arrows) and expected fragment length. Right: Signals corresponding to 2.6 kb for wildtype, 2.4 kb for the deletion, and 2.1 kb as well as 2.4 kb for the complementation were obtained as predicted. (**c**) Southern hybridization of *MEK2* single and double deletion and complementation strains with *Nru*I. Left: Scheme of restriction sites (arrows) and expected fragment length. Right: Signals corresponding to 5.2 kb for WT, 7.1 kb for the deletion, and 5.3 kb as well as 7.1 kb for the complementation were obtained as predicted.

| **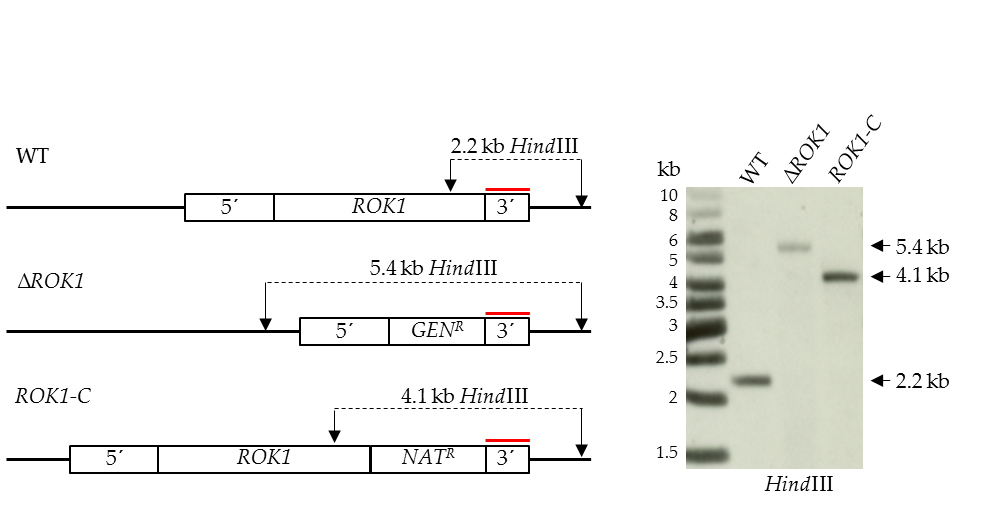** |
| --- |

**Figure S4. Southern hybridization of *V. dahliae ROK1* deletion and *ROK1-C* complementation strains.** *V. dahliae* wildtype (WT), *ROK1* deletion (∆*ROK1*), and the complementation strain harboring *ROK1* under control of the native promoter at the endogenous locus (*ROK1-C*) were tested. *Hind*III was used to cut genomic DNA and the *ROK1* 3′ flanking region served as a probe (red line). Left: Scheme of restriction sites (arrows) and expected fragment length. *GEN^R^*: geneticin resistance cassette with *trpC* promoter and *trpC* terminator; *NAT^R^*: nourseothricin resistance cassette with *gpdA* promoter and a *trpC* terminator. Right: Signals corresponding to 2.2 kb for wildtype, 5.4 kb for the deletion and 4.1 kb for the endogenous complementation strains were obtained as predicted.

|  | 1 | ATG GAG TCT TGG GAG CAC TCC ACC ACA CCA | 30 |
| --- | --- | --- | --- |
|  | 31 | ATG ATC AAG TTC GAG GAC TCG CCA GCC GAG | 60 |
|  | 61 | TCT TTC GTC TCG ACA CCA GGC GAC ATG TAC | 90 |
|  | 91 | CCG TCA CTC TTC CCA GAG TCC GCC TCC CCC | 120 |
|  | 121 | AAC ACC CTC GAT CCT TCC AAC ATG ATG AGC | 150 |
|  | 151 | CCT TCC TCA CCC CAA GAC CTC ACC ATT GCC | 180 |
|  | 181 | GAC ACG GAT ATG CCT CTC TCC GAG GCT TCC | 210 |
|  | 211 | GCC GGC GAC AAG AAG GGG TCC AAG AAG CGC | 240 |
|  | 241 | AAG TCC TGG GGT CAG GTC CTT CCC GAG CCC | 270 |
|  | 271 | AAG ACC AAC TTG CCG CCC AGG AAA CGA GCC | 300 |
|  | 301 | AAG ACT GAG GAT GAG AAG GAG CAG CGT CGT | 330 |
|  | 331 | GTG GAA CGC GTT CTG CGC AAC CGC CGT GCT | 360 |
|  | 361 | GCC CAG TCT TCG AGG GAG CGC AAG AGG CTC | 390 |
|  | 391 | GAG GTT GAG GCC CTC GAG ATG AAG AAC AAG | 420 |
|  | 421 | GAG CTC GAG ACT GCC CTG AAC CAC GCA CAA | 450 |
|  | 451 | CAG GCG AAC GCT AGG TTG ATG GAG GAG CTT | 480 |
|  | 481 | ACC AAG TTC CGC CGT GGT TCC GGT GCC GTC | 510 |
|  | 511 | GCC CGT TCT TCT TCC CCC TTT GAC TCC TTC | 540 |
|  | 541 | CAC AAC AGC AAC TCG GTC ACC CTC TCC CCC | 570 |
|  | 571 | GAG CTG TTC GGC TCT CAA GAC GGC CGC CGG | 600 |
|  | 601 | CCA TCA GTG GCC GAC TCC GAG TCG ACA CTC | 630 |
|  | 631 | GTC GAC GGT TTG ATG GCG GCC TCC AAG TCC | 660 |
|  | 661 | GCC GCG ACC GTC AAC CCC GCC TCC CTC TCG | 690 |
|  | 691 | CCC GCC CTC ACC CCC GTC CCC GAG ACG GAT | 720 |
|  | 721 | GAG ACC AGC GCC CAA CAA GAA GCT GCC GTG | 750 |
|  | 751 | GCC GCC CCT TCC CCT GTC GCC CTT TCC TCC | 780 |
|  | 781 | GAC GTG ACA CAA CGT CCT GCC GTG TCG GTC | 810 |
|  | 811 | GGA GGA AAT GCC TCA GTC GTG GGT GGC CTC | 840 |
|  | 841 | GCA GAC TTC CCT GCA CCC AAC ATG GAC TTT | 870 |
|  | 871 | GTA CCT TCA GCT TCA GAT GCT CAT GAT CAC | 900 |
|  | 901 | TTC CTC GGC GGT CAT TTC AGC GTG TCA GAG | 930 |
|  | 931 | GCC TTT GAT GCA GAT CGC TAT GTC CTT GAG | 960 |
|  | 961 | AGC GGG CTT CTC TCT TCC CCC AAC TCA GTC | 990 |
|  | 991 | GAT TAT GAC AAC GAT ATT ATG GCT GGT GAC | 1020 |
|  | 1021 | TCG TCC GCG TTC GCA TCC GCG TTC AAC TTC | 1050 |
|  | 1051 | GAC ATG GAC GAG TTC CTC AAC GAT GAG GCC | 1080 |
|  | 1081 | AGC GCA GCC GCC ACT GAC GCG TCA GCA GCG | 1110 |
|  | 1111 | GAG AAC AGC GCA GCG GAC CCG GAC TAC GGC | 1140 |
|  | 1141 | CGC CGT GCC CTT AAC CCT GAG ACT CAA GTC | 1170 |
|  | 1171 | TCT TCA GAA AAT CCT AAC CTG CAG CCC CAA | 1200 |
|  | 1201 | TCT GGC GCG TCC ACT TAT GGA TGC GAC GAT | 1230 |
|  | 1231 | GGA GGC ATT GCG GTT GGT GTC TGA | 1254 |

**Figure S5: The cDNA sequence of *V. dahliae* JR2 *HAC1^i^.*** The *V. dahliae HAC1^i^* sequence with 1254 nt was obtained by RNA extraction and cDNA synthesis from mycelium of wildtype cultures grown in 50 ml SXM (1 × 10^7^ spores) for 4 d shaking at 25 °C and subsequent supplementation with 3 mM DTT for 3 h. *HAC1^i^* was amplified using primers JST171/JST174 and fully sequenced.

| 1 | MESWEHSTTP | MIKFEDSPAE | SFVSTPGDMY | PSLFPESASP | 40 |
| --- | --- | --- | --- | --- | --- |
| 41 | NTLDPSNMMS | PSSPQDLTIA | DTDMPLSEAS | AGDKKGSKKR | 80 |
| 81 | KSWGQVLPEP | KTNLPPRKRA | KTEDEKEQRR | VERVLRNRRA | 120 |
| 121 | AQSSRERKRL | EVEALEMKNK | ELETALNHAQ | QANARLMEEL | 160 |
| 161 | TKFRRGSGAV | ARSSSPFDSF | HNSNSVTLSP | ELFGSQDGRR | 200 |
| 201 | PSVADSESTL | VDGLMAASKS | AATVNPASLS | PALTPVPETD | 240 |
| 241 | ETSAQQEAAV | AAPSPVALSS | DVTQRPAVSV | GGNASVVGGL | 280 |
| 281 | ADFPAPNMDF | VPSASDAHDH | FLGGHFSVSE | AFDADRYVLE | 320 |
| 321 | SGLLSSPNSV | DYDNDIMAGD | SSAFASAFNF | DMDEFLNDEA | 360 |
| 361 | SAAATDASAA | ENSAADPDYG | RRALNPETQV | SSENPNLQPQ | 400 |
| 401 | SGASTYGCDD | GGIAVGV |  |  | 417 |

**Figure S6: The amino acid sequence of *V. dahliae* JR2 Hac1.** The deduced protein sequence from *HAC1^i^* is 417 aa in length. Red: NLS predicted by cNLS Mapper (94–105 aa); Blue: N-terminal basic-leucine zipper domain (bZIP, PS50217; 107–164 aa).

| 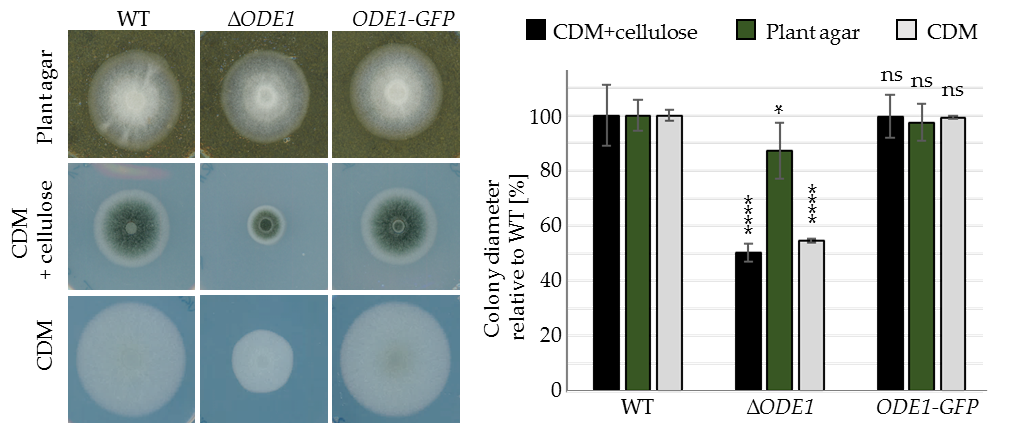 |
| --- |

**Figure S7.** ***V. dahliae* ∆*ODE1* has only a minor effect on vegetative growth on plant agar.** Vegetative growth of *V. dahliae* wildtype (WT), *ODE1* deletion (∆*ODE1*), and complementation (*ODE1-GFP*) strains was quantified 11 d after spot inoculation. Δ*ODE1* displays about 50% decreased growth on CDM with cellulose or sucrose as carbon source. Plant agar partially complements the growth defect to about 90% relative to wildtype. Mean values and standard deviations relative to wildtype are shown. Significant differences to wildtype were calculated (* *p* < 0.05; **** *p* = 0; ns = non-significant; *n* ≥ 3).

| **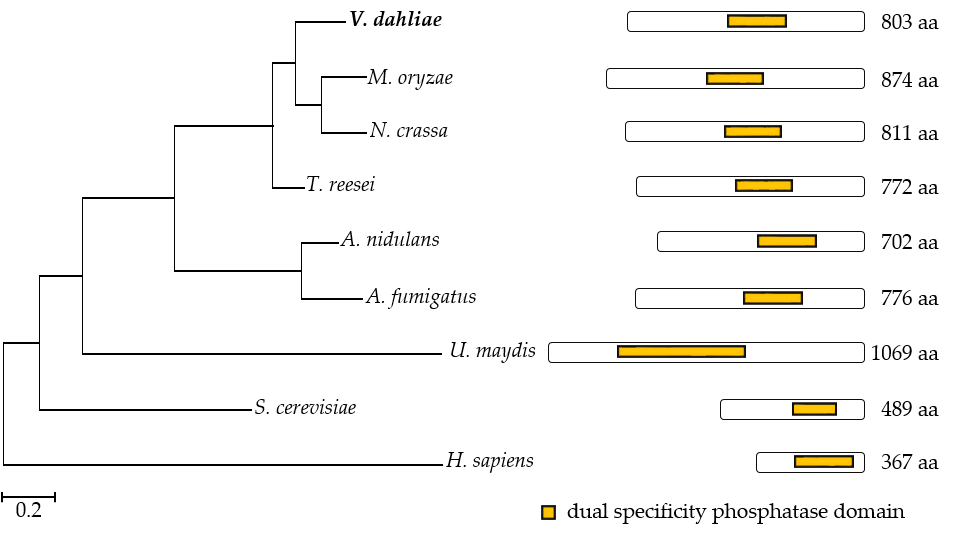** |
| --- |

**Figure S8. Phylogeny of Rok1-like phosphatases.** Relations of Rok1-like phosphatases are depicted in a phylogenetic tree with *Verticillium dahliae* (*VDAG_JR2_Chr7g08960a*), *Magnaporthe oryzae* (XP_003712767.1), *Neurospora crassa* (XP_962856.1), *Trichoderma reesei* (XP_006961240.1), *Aspergillus nidulans* (XP_662148.1), *Aspergillus fumigatus* (XP_749411.1), *Ustilago maydis* (UMAG_03701), *Saccharomyces cerevisiae* (NP_014345), *Homo sapiens* (NP_004408.1) sequences (ClustalW algorithm, scale bar = average number of amino acid substitutions per site). Rok1-like proteins with dual-specificity phosphatase domains (IPR020422, yellow) are shown.

| **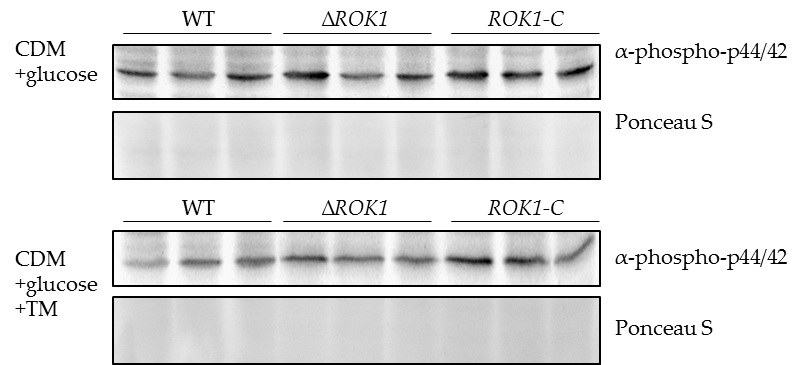** |
| --- |

**Figure S9. Vmk1 phosphorylation is unaffected upon deletion of *ROK1* in *V. dahliae*.** Phosphorylated Vmk1 was visualized in protein extracts obtained from wildtype, *ROK1* deletion (∆*ROK1*), or *ROK1* complementation (*ROK1-C*) strains grown in CDM with glucose with or without supplemented tunicamycin (TM, 1 µg/mL) with a phospho-p44/42 antibody specifically recognizing the phosphorylated TEY motif of MAPKs. The Ponceau S-stained membrane served as loading control.

### *Supplementary Tables*

**Table S1:** **Verticillium strains constructed and used in this study.**

| Strain | Description | Reference |
| --- | --- | --- |
| JR2/ WT | *Solanum lycopersicum* isolate | [67] |
| WT *OE-GFP^HYG^*  (VGB45) | *^p^gpdA:GFP:trpC^t^:^p^gpdA:HYG^R^:trpC^t^* | [69] |
| ∆*HAM5*  (VGB279/ VGB280) | ∆*HAM5::^p^gpdA:NAT^R^:trpC^t^* | This study |
| ∆*ODE1*  (VGB331/ VGB332) | ∆*ODE1:: ^p^gpdA:NAT^R^:trpC^t^* | This study |
| ∆*VMK1*  (VGB335/ VGB336) | ∆*VMK1::^p^gpdA:HYG^R^:trpC^t^* | This study |
| ∆*MEK2*  (VGB337/ VGB338) | ∆*MEK2::^p^gpdA:HYG^R^:trpC^t^* | This study |
| ∆*HAM5∆MEK2*  (VGB346) | ∆*MEK2::^p^gpdA:HYG^R^:trpC^t^; ∆HAM5::^p^gpdA:NAT^R^:trpC^t^* | This study |
| ∆*ODE1 OE-GFP*  (VGB357) | ∆*ODE1:: ^p^gpdA:NAT^R^:trpC^t^; ^p^gpdA:GFP:trpC^t^:^p^gpdA:HYG^R^:trpC^t^* | This study |
| *ODE1-GFP*  (VGB358/ VGB359) | ∆*ODE1::^p^gpdA:NAT^R^:trpC^t^::^p^ODE1:ODE1:GFP:^p^gpdA:HYG^R^:trpC^t^:ODE1^t^* | This study |
| ∆*HAC1*  (VGB371/ VGB372) | ∆*HAC1::^p^gpdA:HYG^R^:trpC^t^* | This study |
| ∆*HAC1 OE-GFP* (VGB380) | ∆*HAC1::^p^gpdA:HYG^R^:trpC^t^; ^p^gpdA:GFP:trpC^t^:^p^gpdA:NAT^R^:trpC^t^* | This study |
| *HAC1-C*  (VGB382) | ∆*HAC1::^p^gpdA:HYG^R^:trpC^t^; ^p^HAC1:HAC1:HAC1^t^:^p^gpdA:NAT^R^:trpC^t^* | This study |
| *MEK2-C*  (VGB388/ VGB389) | ∆*MEK2::^p^gpdA:HYG^R^:trpC^t^; ^p^MEK2:MEK2:MEK2^t^:^p^gpdA:NAT^R^:trpC^t^* | This study |
| WT *OE-GFP^NAT^* (VGB392) | *^p^gpdA:GFP:trpC^t^:^p^gpdA:NAT^R^:trpC^t^* | This study |
| *VMK1-C*  (VGB413) | ∆*VMK1::^p^gpdA:HYG^R^:trpC^t^; ^p^VMK1:VMK1:VMK1^t^: ^p^gpdA:NAT^R^:trpC^t^* | This study |
| *HAM5-C*  (VGB415) | ∆*HAM5::^p^gpdA:NAT^R^:trpC^t^; ^p^HAM5:HAM5:HAM5^t^:^p^gpdA:HYG^R^:trpC^t^* | This study |
| ∆*HAM5*∆*VMK1*  (VGB417) | ∆*VMK1::^p^gpdA:HYG^R^:trpC^t^; ∆HAM5::^p^gpdA:NAT^R^:trpC^t^* | This study |
| *HAC1^u^-HA*  (VGB439/ VGB440) | ∆*HAC1::^p^gpdA:HYG^R^:trpC^t^; ^p^HAC1:HAC1^u^:HA:HAC1^t^:^p^gpdA:NAT^R^:trpC^t^* | This study |
| *HAC1^i^-HA*  (VGB437/ VGB438) | ∆*HAC1::^p^gpdA:HYG^R^:trpC^t^; ^p^HAC1:HAC1^i^:HA:HAC1^t^:^p^gpdA:NAT^R^:trpC^t^* | This study |
| JR2 *Histone-RFP*  (VGB477) | *^p^gpdA:H2B:RFP:trpC^t^:^p^gpdA:GEN^R^:trpC^t^* | This study |
| *ODE1-GFP*  *Histone-RFP*  (VGB493/ VGB494) | ∆*ODE1::^p^gpdA:NAT^R^:trpC^t^::^p^ODE1:ODE1:GFP:^p^gpdA:HYG^R^:trpC^t^:ODE1^t^; ^p^gpdA:H2B:RFP:trpC^t^:^p^gpdA:GEN^R^:trpC^t^* | This study |
| ∆*ROK1*  (VGB532) | ∆*ROK1::^p^trpC:GEN^R^:trpC^t^* | This study |
| *ROK1-C*  (VGB548) | ∆*ROK1::^p^trpC:GEN^R^:trpC^t^; ^p^ROK1:ROK1:^p^gpdA:NAT^R^:trpC^t^:ROK1^t^* | This study |

*^p^*: promoter, *^t^*: terminator, *NAT^R^*: nourseothricin resistance marker, *GEN^R^*: geneticin resistance marker, *HYG^R^*: hygromycin B resistance marker, two VGB numbers for one genotype indicate two independent transformants.

**Table S2: Primers used in this study.**

| Primer name | Primer sequence  (5′🡪 3′) | Length  (-mer) | Overhang to |
| --- | --- | --- | --- |
| JST76b | **GTA TGT TGT GTG GAA** AGC ACG GAG CAG AGA CCA | 33 | pPK2 |
| JST77a | GGT TCT GGT ACA CGA CGA GC | 20 | - |
| JST110 | **GTA TGT TGT GTG GAA** CCG CGA GGG TTG GAG AGG | 33 | pME4564 |
| JST111 | **ACC GGT CAC TGT ACA** GAC GGG CCT GAT ATT CTT TCG A | 37 | *^p^gpdA* |
| JST112 | **AGG TAA TCC TTC TTT** GTG GCC GTC TTT TCA CAG GC | 35 | *trpC^t^* |
| JST113 | **CAC AGT ACA CGA GGA** TCT CGT CCG GAC TGA TCC AA | 35 | pME4564 |
| JST127 | **CGT ATG TTG TGT GGA** AGG ATG GCC AAT GTG GAT TTG AT | 38 | pME4564 |
| JST128 | **CAC CGG TCA CTG TAC** AGG TAC TGG TGG CTC TTG GGA | 36 | *^p^gpdA* |
| JST129 | **GAG GTA ATC CTT CTT** TTC GGA TTG GAC AGT AGA CAA GTT TG | 41 | *trpC^t^* |
| JST130 | **CAC AGT ACA CGA GGA** CGC GCA CAG TTA CAC TTC ATA CTC T | 40 | pME4564 |
| JST137 | **TCC ACA TTG GCC ATC** CTT CCA CAC AAC ATA CGA GCC G | 37 | ^p^*ODE1* |
| JST138 | **AGT GTA ACT GTG CGC** GTC CTC GTG TAC TGT GTA AGC | 36 | *ODE1^t^* |
| JST171 | ATG GAG TCT TGG GAG CAC TC | 20 | - |
| JST172 | TCA GAC ACC AAC CGC AAT | 18 | - |
| JST174 | TCA GCG AAA GCG CAC TC | 17 | - |
| JST177 | AAA GAA GGA TTA CCT CTA AAC AAG TGT | 27 | - |
| JST178 | **GGT ACC GAG CTC GAT** TTA CTT GTA CAG CTC GTC CA | 35 | *^p^gpdA* |
| JST179 | **ACC ACC GCT ACC ACC** CTG CTC ATC CGT ACG GC | 32 | linker C-terminal *GFP* |
| JST180 | **ATT CTT AAT TAA GAT** GGA TGG CCA ATG TGG AT | 32 | pPK2 |
| JST184 | **GTA TGT TGT GTG GAA** CGA GTG GAG ATG TGG AGT | 33 | pME4815 |
| JST185 | **ACC GGT CAC TGT ACA** TGG CAT GCG GAG AGA C | 31 | pME4815 |
| JST186 | **ATT CTT AAT TAA GAT** GAC AAG AGT CAA GCC CAC | 33 | pME4564 |
| JST187 | **AGA TCC CCG GGT ACC** GAT GGA CGA AGC GAC TC | 32 | *^p^gpdA* |
| JST188 | **AGG TAA TCC TTC TTT** TTT GAT TTT TAT CAT GAT GAC GGC | 39 | *trpC^t^* |
| JST189 | **AGG ACT TCT AGA AGG** TCC AGC TCC AAA TCA ATT AAC C | 37 | pME4564 |
| JST211 | **GGT CAC TGT ACA GAT** GGG ACT CGT ACC ATG TTT C | 34 | *trpC^t^* |
| JST212 | **TGT TGT GTG GAA GAT** ACT AAG TAC TGG TTG TGG CTG AC | 38 | pME4815 |
| JST213 | **GGT CAC TGT ACA GAT** AGG CTT GGA GAT GAC GAG | 33 | *^p^gpdA* |
| JST216 | **TGT TGT GTG GAA GAT** GAC AAG AGT CAA GCC CAC | 33 | pME4564 |
| JST243 | **TGT TGT GTG GAA GAT** AGC ACG GAG CAG AGA CCA | 33 | pME4815 |
| JST244 | **GGT CAC TGT ACA GAT** CGA ACC GGT GAT GGA TAC G | 34 | *^p^gpdA* |
| JST245 | **ATT CTT AAT TAA GAT** CTG CTC CTA TTC GGC TCC | 33 | pPK2 |
| JST246 | **GGT ACC GAG CTC GAT** TCT CGT CCG GAC TGA TCC | 33 | pPK2 |
| JST253 | GAC GTT AAC TGA TAT TGA AGG AGC AC | 26 | - |
| JST254 | AAC CCA GGG GCT GGT GA | 17 | - |
| JST266 | **CTA TCC GCC GCT AGC GTA ATC GGG CAC ATC GTA TGG GTA GCC GCC GCT** GAC ACC AAC CGC AAT GC | 65 | *HA* tag |
| JST267 | **CTA TCC GCC GCT AGC GTA ATC GGG CAC ATC GTA TGG GTA GCC GCC GCT** GCG AAA GCG CAC TCG T | 64 | *HA* tag |
| JST268 | CTA TCC GCC GCT AGC GTA | 18 | - |
| JST269 | **TGT TGT GTG GAA GAT** GCT GAG GTC ATG GCT GAC | 33 | pME4564 |
| JST270 | **CTC CCA AGA CTC CAT** TTT GGA CGG CTT TGT GTG | 33 | *HAC1* |
| JST271 | **GCT AGC GGC GGA TAG** GGG CTG TGA GAA TCG GGT | 33 | *HA* |
| JST272 | **GGT CAC TGT ACA GAT** GGG ACT CGT ACC ATG TTT CA | 35 | *trpC^t^* |
| JST273 | **GCT AGC GGC GGA TAG** TTT GAT TTT TAT CAT GAT GAC GG | 38 | *HA* |
| JST290 | TTC AGA AAA TCC TAA CCT GCA G | 22 | - |
| JST291 | ACA CCA ACC GCA ATG CCT | 18 | - |
| JST314 | **ATT CTT AAT TAA GAT** CTA GAT GTG CGC GAC CAA G | 34 | pME4564 |
| JST315 | **ATA TCA GTT AAC GTC** GAT TCA CAA CTG GCG CGA | 33 | *^p^trpC* |
| JST316 | **ACC AGC CCC TGG GTT** GAT ATG AAG ATG TTG AGC TGA | 36 | *trpC^t^* |
| JST317 | **AGG ACT TCT AGA AGG** AGG AGT TTC GAT ATA GCC G | 34 | pME4564 |
| JST319 | **ACC GGT CAC TGT ACA** TCA AAG GAT TTC GTC GAT GGA C | 37 | *^p^gpdA* |
| JST325 | GGA GAA GGA GGC TGT GAG TAT T | 22 | *^-^* |
| JST326 | CCG TTC TCC TCG AGC CAG | 18 | *^-^* |
| JS-V5 | **CGT ATG TTG TGT GGA A**AC TAA GTA CTG GTT GTG GCT GAC | 39 | pPK2 |
| JS-V6 | **AAG ATC CCC GGG TAC C**TT TGG GTG ATG TGC GTG G | 34 | *^p^gpdA* |
| JS-V7 | **ACA ACC AGT ACT TAG T**TT CCA CAC AAC ATA CGA G | 34 | *^p^MEK2* |
| JS-V8 | **ACG CAC ATC ACC CAA A**GG TAC CCG GGG ATC TTT C | 34 | *^p^MEK2* |
| JS-V9 | **TCC TTC TTT CTA GAA G**TT GAA CAG GCC TGT CTG G | 34 | pME4821 |
| JS-V10 | **ACA CAG TAC ACG AGG A**AG GCT TGG AGA TGA CGA G | 34 | pME482 |
| JS-V11 | **CGT CAT CTC CAA GCC T**TC CTC GTG TAC TGT GTA AG | 35 | *MEK2^t^* |
| JS-V12 | **AGA CAG GCC TGT TCA A**CT TCT AGA AAG AAG GAT TAC CTC | 39 | *MEK2^t^* |
| JS-V21 | **GAG GTA ATC CTT CTT T**GG TGG CAG TGG CAG TGG | 33 | pPK2 |
| JS-V22 | **ACA CGA GGA CTT CTA G**CG AAC CGG TGA TGG ATA CGT T | 37 | pPK2 |
| JS-V23 | **ATC CAT CAC CGG TTC G**CT AGA AGT CCT CGT GTA CTG T | *37* | *VMK1^t^* |
| JS-V24 | **CAC TGC CAC TGC CAC C**AA AGA AGG ATT ACC TCT AAA CAA GT | 41 | *VMK1^t^* |
| JT1 | **CTC TAG AGG ATC CCC** TGT ACA GTG ACC GGT GAC TC | 35 | pCOM |
| JT2 | **TCG AGC TCG GTA CCC** ACC TCT AAA CAA GTG TAC CTG T | 37 | pCOM |
| ML1 | TTC CAC ACA ACA TAC GAG CC | 20 | - |
| ML2 | TCC TCG TGT ACT GTG TAA GC | 20 | - |
| ML5 | TGT ACA GTG ACC GGT GAC TCT T | 22 | - |
| ML6 | **TCC CGC GGT CGG CAT** CTA CTT CAG GGG CAG GGC ATG CT | 38 | *trpC^t^* |
| ML7 | **TGA GCA TGC CCT GCC** CCT GAA GTA GAT GCC GAC CGC G | 37 | *NAT^R^* |
| ML8 | AAA GAA GGA TTA CCT CTA AAC AA | 23 | - |
| ML9 | TGT ACA GTG ACC GGT GAC | 18 | - |
| PC4 | TGT ACA GTG ACC GGT GAC TC | 20 | - |
| pJet1.2 reverse | AAG AAC ATC GAT TTT CCA TGG CAG | 24 | - |
| RH523 | **ACG TCC TCG GAG GAG GCC AT**G GTG ATG TCT GCT CAA GCG | 39 | *RFP* |
| RH524 | CCG CTT GAG CAG ACA TCA CC**A TGG CCT CCT CCG AGG AC** | 38 | *^p^gpdA* |
| RH525 | **GGC ATA CCA CCG CTA CCA CC**G GCG CCG GTG GAG TGG C | 37 | Linker, *H2B* |
| RH526 | **GCG CCG GTG GTA GCG GTG GT**A TGC CCC CCA AGG CCG C | 37 | Linker, *RFP* |
| RH527 | **TCC CGC GGT CGG CAT CTA CT**T TAT TTC GTG GAC GAG GAA TAC | 42 | *trpC^t^* |
| RH528 | **ATT CCT CGT CCA CGA AAT AA**A GTA GAT GCC GAC CGC GG | 38 | *H2B* |
| RH529 | TCT AGA AAG AAG GAT TAC CTC T | 22 | - |
| RH530 | **AGT TCT AGA** TGT ACA GTG ACC GGT GAC TC | 29 | Restriction site |
| RO3 | GGT ACC CGG GGA TCT TTC G | 19 | - |
| SAB16 | GGT GGT AGC GGT GGT ATG | 18 | - |
| SAB52 | **GTA TGT TGT GTG GAA gat atc** TGT ACA GTG ACC GGT GAC | 39 | pME4564 |
| SAB53 | **CAC AGT ACA CGA GGA** AAA GAA GGA TTA CCT CTA AAC AA | 38 | pME4564 |
| SZ9 | AAC ACC CAG AAC AAG ATG CGC | 21 | - |
| SZ10 | GCT TGA CCT TGA GAT CCT TG | 20 | - |
| SZ11 | TGC ATT CTT GGC AAG AGA TGT GTG | 24 | - |
| SZ12 | AGC TTG TTA TCC TTG TCC TCG GT | 23 | - |

Bold: overhangs for fusion PCR, Seamless and FastCloning, restriction sites, or overhang for fusion of linker or tag, *^p^*: promoter, *^t^*: terminator.

**Table S3: Plasmids constructed and used in this study.**

| Plasmid | Description | Reference |
| --- | --- | --- |
| pCOM | *^p^trpC:GEN^R^:trpC^t^, KAN^R^,* left and right border for ATMT | [73] |
| pGreen2 | *^p^gpdA:HYG^R^:trpC^t^*; *^p^gpdA:GFP:trpC^t^*; *KAN^R^*; left and right border for ATMT | [69] |
| pJet1.2 | Cloning vector with *AMP^R^* | Thermo Fisher Scientific, Waltham, MA, USA |
| pPK2 | Cloning vector with *KAN^R^* and *HYG^R^*; left and right border for ATMT | [70] |
| pKO2.0 | Cloning vector with *KAN^R^* and *NAT^R^*: left and right border for ATMT | [71] |
| pME3857 | *^p^gpdA:mRFP:H2A* | [72] |
| pME4564 | Cloning vector with *KAN^R^* and *HYG^R^*; left and right border for ATMT | [71] |
| pME4815 | *^p^gpdA:NAT^R^:trpC^t^* in pME4564, *KAN^R^* | [71] |
| pME4819 | *^p^gpdA:GFP:trpC^t^* in pME4815 | This study |
| pME4820 | *^p^HAM5:^p^gpdA:NAT^R^:trpC^t^*: *HAM5^t^* in pME4564 | This study |
| pME4821 | *^p^MEK2* in pPK2 | This study |
| pME4822 | *^p^MEK2:^p^gpdA:HYG^R^:trpC^t^*:*MEK2^t^* in pPK2 | This study |
| pME4823 | *HAM5^t^* in pPK2 | This study |
| pME4824 | *VMK1*^t^ in pPK2 | This study |
| pME4825 | *^p^VMK1:^p^gpdA:HYG^R^:trpC^t^*:*VMK1^t^* in pPK2 | This study |
| pME4826 | *^p^MEK2:MEK2:MEK2^t^* in pME4815 | This study |
| pME4827 | *^p^VMK1:VMK1:VMK1^t^* in pME4815 | This study |
| pME4828 | *^p^HAM5:HAM5:HAM5^t^:^p^gpdA:HYG^R^:trpC^t^* in pPK2 | This study |
| pME4830 | *^p^HAC1:^P^gpdA:HYG^R^:trpC^t^*:*HAC1^t^* in pME4564 | This study |
| pME4831 | *^p^HAC1:HAC1:HAC1^t^* in pME4815 | This study |
| pME4832 | *HAC1^u^:HA* in pJet1.2 | This study |
| pME4833 | *HAC1^i^:HA* in pJet1.2 | This study |
| pME4834 | *^p^HAC1:HAC1^u^:HAC1^t^* in pME4815 | This study |
| pME4835 | *^p^HAC1:HAC1^i^:HAC1^t^* in pME4815 | This study |
| pME4836 | *^p^ODE1:^p^gpdA:NAT^R^:trpC^t^*:*ODE1^t^* in pME4564 | This study |
| pME4837 | *ODE1*^t^ in pPK2 | This study |
| pME4838 | *^p^ODE1:ODE1:GFP: ^p^gpdA:HYG^R^:trpC^t^:ODE1^t^* in pME4837 | This study |
| pME4973 | *^p^gpdA:RFP:H2B:trpC^t^* in pJet1.2 | This study |
| pME4975 | *^p^gpdA:RFP:H2B:trpC^t^* in pPK2 | This study |
| pME4976 | *^p^gpdA:RFP:H2B:trpC^t^* in pCOM | This study |
| pME5092 | *^p^ROK1:^p^trpC:GEN^R^:trpC^t^:ROK1^t^* in pME4564 | This study |
| pME5093 | *^p^ROK1:ROK1:^p^gpdA:NAT^R^:trpC^t^:ROK1^t^* in pME4564 | This study |

*AMP^R^*; ampicillin resistance marker, ATMT: *Agrobacterium tumefaciens-*mediated transformation, *GEN^R^*: geneticin resistance marker, *HYG^R^*: hygromycin B resistance marker, *KAN^R^*: kanamycin resistance marker, *NAT^R^*: nourseothricin resistance marker, *^p^*: promoter, *^t^*: terminator
